# Behavioral and neural network abnormalities in human APP transgenic mice resemble those of *App* knock-in mice and are modulated by familial Alzheimer’s disease mutations but not by inhibition of BACE1

**DOI:** 10.1186/s13024-020-00393-5

**Published:** 2020-09-14

**Authors:** Erik C. B. Johnson, Kaitlyn Ho, Gui-Qiu Yu, Melanie Das, Pascal E. Sanchez, Biljana Djukic, Isabel Lopez, Xinxing Yu, Michael Gill, Weiping Zhang, Jeanne T. Paz, Jorge J. Palop, Lennart Mucke

**Affiliations:** 1 Gladstone Institute of Neurological Disease, 1650 Owens Street, San Francisco, CA 94158 USA; 2 Department of Neurology and Weill Institute for Neurosciences, University of California, San Francisco, San Francisco, CA 94158 USA; 3 NHC Key Laboratory of Hormones and Development, Tianjin Institute of Endocrinology, Tianjin Medical University Metabolic Diseases Hospital, Tianjin, China

**Keywords:** Alzheimer’s disease, Amyloid, APP, APP-KI, *App*^NL-G-F^, BACE, Behavior, Calbindin, C-Fos, Epilepsy, Epileptiform, I5, Inhibitor, J20, Knock-in, Learning and memory, Oligomers, SWD

## Abstract

**Background:**

Alzheimer’s disease (AD) is the most frequent and costly neurodegenerative disorder. Although diverse lines of evidence suggest that the amyloid precursor protein (APP) is involved in its causation, the precise mechanisms remain unknown and no treatments are available to prevent or halt the disease. A favorite hypothesis has been that APP contributes to AD pathogenesis through the cerebral accumulation of the amyloid-β peptide (Aβ), which is derived from APP through sequential proteolytic cleavage by BACE1 and γ-secretase. However, inhibitors of these enzymes have failed in clinical trials despite clear evidence for target engagement.

**Methods:**

To further elucidate the roles of APP and its metabolites in AD pathogenesis, we analyzed transgenic mice overexpressing wildtype human APP (hAPP) or hAPP carrying mutations that cause autosomal dominant familial AD (FAD), as well as *App* knock-in mice that do not overexpress hAPP but have two mouse *App* alleles with FAD mutations and a humanized Aβ sequence.

**Results:**

Although these lines of mice had marked differences in cortical and hippocampal levels of APP, APP C-terminal fragments, soluble Aβ, Aβ oligomers and age-dependent amyloid deposition, they all developed cognitive deficits as well as non-convulsive epileptiform activity, a type of network dysfunction that also occurs in a substantive proportion of humans with AD. Pharmacological inhibition of BACE1 effectively reduced levels of amyloidogenic APP C-terminal fragments (C99), soluble Aβ, Aβ oligomers, and amyloid deposits in transgenic mice expressing FAD-mutant hAPP, but did not improve their network dysfunction and behavioral abnormalities, even when initiated at early stages before amyloid deposits were detectable.

**Conclusions:**

hAPP transgenic and *App* knock-in mice develop similar pathophysiological alterations. APP and its metabolites contribute to AD-related functional alterations through complex combinatorial mechanisms that may be difficult to block with BACE inhibitors and, possibly, also with other anti-Aβ treatments.

## Background

Extensive genetic evidence suggests that the amyloid precursor protein (APP) is causally involved in the pathogenesis of Alzheimer’s disease (AD) [1–8]. However, the precise mechanisms that underlie this involvement have, if anything, become less certain as the investigation of this protein has advanced from the research bench into clinical trials. Expression of the APP holoprotein or of some of its metabolites has been shown to exert potentially AD-relevant effects in diverse experimental models [9–26]. Neuropathological and biochemical investigations focused most of the attention in this field on the cerebral deposition of amyloid-β (Aβ) peptides [8, 27–29], which are released from APP through cleavage by the β-site APP cleaving enzyme (in brain mostly BACE1) and by the multiprotein complex known as γ-secretase [30, 31]. The accumulation of Aβ in the form of amyloid plaques is a pathological hallmark of AD and has emerged as a good biomarker of disease risk [32].

Despite all the evidence suggesting a causal role of Aβ in AD, including mutations in APP and presenilin 1 and 2 that cause autosomal dominant, early-onset familial AD (FAD) and promote cerebral Aβ accumulation [4], inhibitors of BACE1 or γ-secretase have yielded negative results in clinical trials involving sporadic AD patients with cognitive impairments [33, 34]. Furthermore, peripheral infusions of anti-Aβ antibodies cleared amyloid from brains of AD patients, but had no or only weak effects on cognitive decline [35–37]. The interpretation of these results is complicated by the lack of information on whether any of these anti-Aβ therapies reduced cerebral levels of Aβ oligomers, which may be more bioactive and neurotoxic than amyloid plaques and fibrils [38–47]. Interestingly, at least some neuropathogenic effects of Aβ oligomers require the presence of APP [48], and treatment of APP transgenic mice with some clinically relevant anti-Aβ antibodies failed to reduce Aβ oligomer levels and behavioral deficits, and exacerbated neuronal hyperactivity and premature mortality in these models [49, 50].

In light of these intriguing findings, we set out to reexamine the relationship between AD-relevant functional abnormalities and APP-related measures in mouse models that overexpress human APP (hAPP), Aβ, or both (Table 1). These models included hemizygous transgenic mice with neuronal overexpression of wildtype hAPP (line I5) [24, 51, 52] or FAD-mutant hAPP (line J20) [24, 52–55] on a mouse *App* wildtype (*App*^+/+^) background, and homozygous *App*^NL-G-F^ knock-in mice in which both endogenous *App* alleles have a humanized Aβ sequence and carry three FAD mutations [56]. For brevity, the genetically modified mice from these specific lines will be referred to simply as I5, J20 and KI mice, respectively. For each line, non-transgenic wildtype (WT) C57Bl/6 J mice obtained from the same breedings that gave rise to the genetically modified mice were used as controls.

**Table 1:** Mouse Models Analyzed

Mouse Lines	I5	J20	KI
Genotype	hAPP-I5	hAPP-J20	*App*^NL-G-F^
Genetic Modification	hAPP transgene	hAPP transgene	*App* knock-in
APP mutations^a^	None	Swedish (KM670/671NL) Indiana (V717F)	Humanized Aβ sequence Swedish (KM670/671NL) Arctic (E693G) Beyreuther/Iberian (I716F)
APP Isoforms Expressed	hAPP770 hAPP751 hAPP695	hAPP770 hAPP751 hAPP695	Unknown (presumably mostly APP695)
Promoter	Human *PDGF-β*	Human *PDGF-β*	Mouse *App*
Genetic Background	C57BL/6 J	C57BL/6 J	C57BL/6 J
Original Reference	Mucke et al., 2000	Mucke et al., 2000	Saito et al., 2014

^a^ with reference to APP770; *N/A* not applicable

We focused our behavioral analysis on learning and memory, because these cognitive functions are severely impaired by AD, and our electrophysiological analysis on electroencephalographic (EEG) recordings, because such recordings can be readily obtained also in humans. Indeed, various types of neural network dysfunction have been detected by EEG in AD patients [57–63] and related mouse models [53, 54, 60, 64–68]. We are particularly interested in non-convulsive epileptiform activity, because we and others recently showed that this activity is more prevalent in AD patients than is widely recognized [57, 58, 60, 62, 63], its detection predicts faster cognitive decline in AD [63], it could promote disease progression through multiple mechanisms [69], and the relationship between epileptiform activity and APP/Aβ is a matter of debate [26, 70].

Here we demonstrate that KI mice, which do not overexpress APP, have robust non-convulsive epileptiform activity and that this activity is associated with elevated levels not only of Aβ, but also of BACE1-generated C-terminal APP fragments (β-CTF or C99), particularly in the neocortex. We further illustrate that differences in the extent of epileptiform activity and in deficits in learning and memory among hAPP transgenic and *App* knock-in mice cannot be readily explained by differences in hippocampal or cortical levels of total Aβ or Aβ oligomers. Moreover, treatment with a BACE1 inhibitor did not significantly reduce cognitive and neural network dysfunctions in J20 mice, although it markedly reduced levels of Aβ peptides, Aβ oligomers, C99 and amyloid plaques. Thus, the roles of APP and APP mutations in the pathogenesis of AD appear to be complex and may involve mechanisms that are unlikely to respond to treatments aimed primarily at the production, accumulation or clearance of Aβ and other secretase-generated APP metabolites.

## Materials and methods

### Mice

The mouse models used in this study are summarized in Table 1. I5 and J20 mice were generated in-house and maintained on a C57BL/6 J genetic background [24]. KI mice on a C57BL/6 J background [56] were obtained from Drs. Takashi Saito and Takaomi Saido (RIKEN Brain Science Institute). APP-deficient (*App*^–/–^) mice were obtained from Dr. Charles Weissmann (University of Zurich). For lines I5 and J20, we bred hemizygous transgenic males with WT C57BL/6 J females obtained from Jackson Labs (stock # 000664) to generate hemizygous transgenic mice and WT littermates. For the KI line, we bred hemizygous knock-in males with hemizygous knock-in females to generate homozygous knock-in mice and WT littermates. Groups of genetically modified mice and WT controls were always generated by the breeding schemes described above, although not all genetically modified mice and WT controls compared in individual experiments were raised by the same dam at the same time (littermates). For each experiment and line, experimental and control groups included roughly comparable proportions of males and females, unless indicated otherwise in figure legends. Mouse pups were weaned 3–4 weeks after birth and housed up to 5 per cage. Mice were fed a regular chow diet (PicoLab Rodent Diet 5053, TestDiet), unless indicated otherwise, and maintained on a 12-h light/dark cycle. The survival of mice was carefully monitored from the time they reached 1 month of age until they were sacrificed. All animal experiments were approved by the Institutional Animal Care and Use Committee of the University of California, San Francisco.

### BACEi treatment

The BACEi NB-360 [71, 72] was obtained from Novartis Institutes for BioMedical Research. It was added to bacon flavored chow (S7334, Rodent Diet, Grain-base (5053); Bio-Serv) at 0.5 g/kg chow. Bacon flavored chow lacking NB-360 (S7331, Rodent Diet, Grain-base (5053); Bio-Serv) served as the negative control. Some of the mice analyzed in this study were maintained on either diet starting at 1 month of age and continuing until the time of sacrifice. Treatment periods and doses are specified in the main text or figure legends.

### Isolation and processing of brain tissues

Mice were anesthetized with 2,2,2-tribromoethanol (Avertin), perfused with 0.9% saline, and their hemibrains removed. Hemibrains were either snap frozen on dry ice and stored at -80° C or drop-fixed in 4% paraformaldehyde/phosphate-buffered saline (PBS) for 48 h and immersed in a 30% sucrose/PBS solution for 1–3 days at 4° C. Coronal sections (30 μm thickness) of fixed brain tissues were prepared with a freezing microtome (Leica SM 2000R) and stored for later immunohistochemical analyses in cryoprotectant solution containing 30% ethylene glycol (Fisher Scientific), 30% glycerol (Fisher Scientific), and 40% PBS at -20° C. Hemibrains were snap-frozen and stored at -80° C. For Western blotting and ELISA, they were thawed on ice for 1 h before the hippocampus and cortex were isolated and weighed separately.

### Tissue homogenization and fractionation

For the analysis of overall Aβ levels by ELISA, brain tissues were homogenized in 5 M guanidine buffer [73]. For western blot analysis of CTFs, tissues were homogenized in detergent-free Extraction Buffer 1 (EB1) and Protease inhibitor cocktail set III from the ProteoExtract Transmembrane Protein Extraction kit (EMD Millipore, 71772-3).

For analysis of Aβ oligomer levels, cold PBS homogenization buffer (50 mM NaH_2_PO_4_, 137 mM NaCl, pH 7.4) and protease inhibitor (Complete EDTA-free, Roche) were added at 10:1 volume/weight ratio for hippocampus and 4:1 volume/weight ratio for cortex. Tissues were homogenized with a Storm 24 bullet blender tissue homogenizer (Next Advance) at 4° C for 5 min at setting #6. In pilot experiments, we tested whether Dounce homogenization using glass tubes and 25 strokes on a Glas-Col homogenizer at setting 15 or the use of phosphate-buffered versus Tris-buffered solutions affected the signal in the 3D6/3D6 Meso Scale Discovery (MSD) ELISA (see below). Because these methods yielded comparable results, we used blender homogenization and PBS-based solutions in our study. Homogenized tissues were centrifuged at 200,000 x *g* (Beckman Coulter Optima Max Ultracentrifuge, MLA-130 rotor) for 30 min at 4° C in polycarbonate centrifuge tubes (Beckman Coulter) to pellet insoluble material and Aβ fibrils [47, 74–77]. The supernatant was removed and frozen at -80°  C and later thawed to measure Aβ oligomer levels (PBS-soluble fraction). The pellet was resuspended in cold PBST homogenization buffer (1% Triton X-100, 50 mM NaH_2_PO_4_, 137 mM NaCl, pH 7.4) plus protease inhibitor at the appropriate volume/weight ratio and rehomogenized using the same protocol. The PBST homogenate was centrifuged as above, the supernatant removed and stored at -80° C, and thawed to measure Aβ oligomer levels (PBST-soluble fraction).

For analysis of voltage-gated sodium channel proteins (Na_V_), tissues were homogenized in Pierce RIPA buffer (Thermo Fisher Scientific, 89901) with Complete protease inhibitor (Roche, 13853000) and phosphatase inhibitor cocktail 2 & 3 (Sigma-Aldrich, P5726, P0044).

### Western blot analyses

Protein concentrations were determined by the Bradford assay (Bio-Rad) [78]. For analysis of full-length APP, 20 μg of protein was loaded per lane on 3–8% Tris-Acetate gels (Bio-Rad, 3450130) and transferred onto nitrocellulose membranes for 10 min at 25 V using iBlot 2 (ThermoFisher Scientific, IB21001). For analysis of CTFs, 75 μg of protein per sample was added to 1x Tricine Sample buffer (200 mM Tris-HCl, pH 6.8, 40% glycerol, 2% SDS, and trace amounts of Orange G dye) and 2% β-mercaptoethanol, heated for 10 min at 70° C, loaded on a 16.5% Tris-Tricine gel (Bio-Rad, 3450064), and electrophoresed in freshly-prepared cold 1x Tris-Tricine-SDS running buffer (Bio-Rad, 1610744). CTFs were transferred to a nitrocellulose membrane overnight at 0.15 A and 4° C using a Criterion blotter (Bio-Rad).

Membranes were blocked with 5% non-fat milk in tris-buffered saline (TBS) for 2.5 h at room temperature or overnight at 4° C, and probed with antibodies against APP (22C11), CTF (CT15) and actin for 3 h at room temperature. All primary and secondary antibodies used in this study are listed in Additional File 1: Table S1. After 4 washes alternating between TBS and TBS containing 0.1% Tween 20 (TBST), membranes were incubated for 30 min at room temperature with the appropriate matching secondary antibodies conjugated to IRDye in Odyssey blocking buffer (LI-COR, 927–50000) containing 0.2% Tween 20. Membranes were washed 4x, alternating between TBS and TBST, and imaged with an Odyssey CLx Imager (LI-COR). Signal intensities were quantified with Image Studio version 5.2.5 software (LI-COR).

For analysis of Na_V_ proteins, 15–20 μg of protein was loaded per lane on 4–12% Bis-Tris gels (Thermo Fisher Scientific, WG1402BX10) and transferred onto nitrocellulose membranes at 35 V for 3 h at 4° C using the Criterion blotter (Bio-Rad). Membranes were blocked with 5% non-fat dry milk diluted in TBS for 1 h at room temperature, labeled with primary antibodies (Additional File 1: Table S1) overnight at 4° C, and incubated for 1 h at room temperature with matching secondary antibodies conjugated to HRP. After four 10-min washes, blots labeled with HRP-conjugated antibodies were exposed to Pierce ECL (Thermo Fisher Scientific, 32209) and developed on X-ray film. Images were quantified using Fiji – ImageJ (https://imagej.net/Fiji).

To control for differences in loading, the signal of the protein of interest, e.g., for APP holoprotein or CTF, was divided by the actin signal obtained from the same sample. To control for blot to blot variations in signal strengths, APP/actin and CTF/actin ratios were further divided by the average of the corresponding ratios from two J20 standards from the same blot. These J20 standards consisted of the same two J20 samples that were run on every gel.

### ELISA of overall Aβ levels

Levels of human Aβ_1–x_ and Aβ_1–42_ were determined by ELISA as described [73].

### Quantitation of Aβ oligomer levels by 3D6/3D6 MSD ELISA

We adopted the Aβ oligomer assay developed by Yang and colleagues [47]. It is carried out on an electrochemiluminescence (ECL) platform that uses photon generation and detection to increase sensitivity and dynamic range of the oligomer measurements, and allows for the use of smaller sample volumes compared to traditional ELISA assays [79, 80]. Uncoated 96-well standard MSD MULTI-ARRAY plates were coated with capture antibody (30 μl) at 2 μg/ml in 1x phosphate-buffered saline (PBS) overnight at 4° C. Coating solution was then removed and wells blocked with 3% MSD blocker A (purified bovine serum albumin) solution (150 μl) for 1.5 h at room temperature. Wells were then washed once with PBS containing 0.05% Tween 20 (200 μl) and samples (25 μl) were added to the wells. Plates were shaken at room temperature for 1.5 h, samples removed, and wells washed with PBS containing 0.05% Tween 20 (200 μl × 3). Biotinylated detection antibody was then added to wells at 1 μg/ml in 1% MSD blocker A solution (25 μl) and plates were shaken at room temperature for 1 h. Wells were washed three times (200 μl × 3), followed by addition of streptavidin SULFO-Tag conjugate at 0.5 μg/ml (25 μl/well) and shaking at room temperature for 30 min. Wells were then washed (200 μl × 3), MSD read buffer (150 μl) added to wells, and plates read immediately on an MSD Sector Imager 2400 (Meso Scale Discovery). Curve fitting was performed using the four parameter logistic fit function as implemented in the Meso Scale Discovery Workbench version 4 software. Anti-Aβ capture antibodies used for the Aβ oligomer assay were: 3D6, 82E1 (BSA-free), 26D6, or 6E10 (Additional File 1: Table S1). Detection antibodies were biotinylated in-house except for 82E1-biotin and 6E10-biotin, which were acquired from the same supplier as the primary antibody.

Several peptides were used to validate the assay: Aβ_1–6_, Aβ_1–10_, Aβ_1–17_, Aβ_[− 1]-17 R5G_, Aβ_1–42_, Aβ_1–18_ dimer, and Aβ_1–29_ dimer were synthesized in-house, while Aβ42–1 was obtained from Biopeptide (San Diego, California), Aβ_1–40_ from rPeptide (Bogart, Georgia), Aβ_1–40_ diY dimer crosslinked at tyrosine10 from Dr. Dominic Walsh (Harvard), and Aβ1–40 S26C dimer from AnaSpec (Fremont, California).

### Chemicals and reagents for peptide synthesis

2-(1*H*-Benzotriazol-1-yl)-1,1,3,3-tetramethyluronium hexafluorophosphate (HBTU) was obtained from Peptides International (Louisville, Kentucky). 1-[Bis (dimethylamino)methylene]-1H-1,2,3-triazolo [4,5-b] pyridinium 3-oxid hexafluorophosphate (HATU) and *N*,*N*-diisopropylethylamine (DIEA) were obtained from Applied Biosystems (Foster City, California). Piperidine was from Spectrum (Gardena, California). *N*,*N*-Dimethylformamide (DMF), methylene chloride (DCM), methanol, diethyl ether, and trifluoroacetic acid (TFA) were from Thermo Fisher Scientific (Pittsburg, Pennsylvania). Triisopropylsilane (TIS) and 1,2-Ethanedithiol (EDT) were from Sigma-Aldrich (Saint Louis, Missouri). HPLC-grade acetonitrile (ACN), amino acids, and TentaGel resin (NovaSyn TGR) were from EMD Millipore (Billerica, Massachusetts). Side chain protecting groups were: Arg (Pbf), Asn (Trt), Asp (OtBu), Cys (Trt), Gln (Trt), Glu (OtBu), His (Trt), Lys (Boc), Ser (tBu), Thr (tBu), Trp (Boc), and Tyr (tBu).

### Peptide synthesis and purification

Peptides were synthesized manually on the solid phase (TGR resin, NovaSyn) using standard fluorenylmethyloxycarbonyl (Fmoc) chemistry. Aβ_1–42_ was synthesized using a trityl linker, whereas the shorter Aβ peptides were synthesized using a Rink amide linker. Five-fold molar excess of amino acid activated with HBTU for 90 s was used for each acylation reaction, and reacted with the peptide-resin for 40 min. Fmoc deprotection was performed with 20% piperidine in DMF for 20 min. Fmoc-N-methyl amino acids were activated with HATU and reacted for 40 min. Fmoc deprotection of N-methyl amino acids was performed for 40 min, followed by amino acid activation with HATU and reaction with the peptide-resin for 3 h and second coupling for 13–19 h. After chain assembly was complete, the final Fmoc protecting group was removed and the resin washed with DCM and methanol and dried overnight under vacuum. Side-chain protection and cleavage from the resin was performed for 1 h under argon using 95% TFA, 2.5% H_2_O and 2.5% TIS. If cysteine or tryptophan was present, the cleavage cocktail was 94% TFA, 2.5% H_2_O, 2.5% EDT, and 1% TIS instead. The resin was then filtered and the peptide precipitated with cold diethyl ether, washed twice with ether, dissolved in 1:1 ACN:H_2_O + 0.1% TFA, and lyophilized. Full-length Aβ peptides were not dissolved in ACN:H_2_O, but were purified directly from the precipitated crude material according to Burdick et al. [81]. Peptides were purified by high-pressure liquid chromatography (HPLC) on a 22 × 250 mm C4 reversed-phase column (Grace Vydac) using a gradient of B (ACN + 0.08% TFA) vs. A (H_2_O + 0.1% TFA). Fractions containing the target peptide were collected, pooled, and lyophilized. Purified peptides were analyzed on an Agilent 1200 analytical HPLC system using either a 2.1 × 50 mm C4 column (Grace Vydac) or a 4.6 × 150 mm C18 column (Agilent) and the same binary solvent system used for preparative HPLC. Peptide masses were determined with a nanoflow electrospray ionization MS/MS orbitrap system (Velos Pro, Thermo Fisher Scientific). Disulfide dimers were prepared by dissolving the monomeric peptide in oxidation buffer (6 M guanidine hydrochloride, 0.2 M Na_2_HPO_4_, and 1% DMSO, pH 8.0) to ~ 12 mM and, after adjusting the pH to 7.5, stirring the solution open to room air for 7 days. The disulfide dimers were then purified by HPLC as described above.

### Preparation of Aβ oligomers

Aβ_1–40_ oligomers (ADDLs) were prepared as described [82] except that a buffer of 50 mM NaH_2_PO_4_ and 137 mM NaCl (pH 7.4) was used instead of F12 medium. Aβ_1–42_ oligomers were prepared by dissolving synthetic lyophilized peptide in 0.1 M NaOH to a concentration of 2 mM, then diluting to a final peptide concentration of 100 μM in 50 mM NaH_2_PO_4_ and 137 mM NaCl (pH 7.4) and incubating for 4 h at room temperature [83]. The preparations were then aliquoted and stored at -80° C.

### Size-exclusion chromatography (SEC)

Solutions were injected onto a Superdex 75 10/300 GL column (GE Healthcare) and eluted over one column volume with a solution containing 50 mM NaH_2_PO_4_ and 137 mM NaCl (pH 7.4). Peaks were analyzed by UV detection at 280 nm, and 1-ml fractions collected. Protein concentration in each peak was determined by UV absorption at 280 nm using the appropriate calculated extinction coefficient for each peptide. Apparent molecular weight was estimated using both protein and sparsely-branched dextran standards [84–86]. Dextran analytical standards were from Sigma (31430) and protein standards from Bio-Rad (151–1901).

### Immunoprecipitation

Antibodies were incubated with brain homogenates (cortex plus hippocampus) prepared in PBS homogenization buffer as described above at a 1:40 antibody to homogenate protein ratio (total volume 150–200 μl) at 4° C with occasional mixing for 1.5 h. Magnetic protein G beads (MagnaBind, Thermo Fisher Scientific) were washed twice with PBS homogenization buffer and added in a 3:1 protein G to antibody ratio (for the sham immunoprecipitation, protein G beads were added using a volume equivalent to that required for the 82E1 antibody). The solution was then incubated at 4° C for 2 h with occasional mixing. After brief centrifugation at low *g* to pellet the magnetic beads, the supernatant was removed and analyzed by the 3D6/3D6 MSD ELISA and western blotting as described above. The beads were then washed twice with homogenization buffer, and protein eluted by heating to 95° C for 10 min in 1x LDS sample buffer for western blot analysis. The same procedure was repeated for brain homogenates prepared in PBST homogenization buffer, using PBST homogenization buffer for washing rather than PBS homogenization buffer. To use precipitation antibodies consistently for PBS and PBST preparations, the same volume of antibody was applied. Because the PBST homogenates had a higher protein concentration, the precipitation antibody to homogenate protein ratio was less than 1:40 for the PBST experiments.

### Immunohistochemistry

For immunostaining and Thioflavin S staining of Aβ deposits, brain sections were blocked at room temperature, first with 0.3% Sudan Black (Sigma-Aldrich, 199664-25G) in 70% ethanol and PBS for 15 min and then with 10% normal goat serum (Jackson ImmunoResearch Laboratories, 005–000-121) in PBS for 1 h to reduce autofluorescence and non-specific antibody binding. Sections were incubated overnight at room temperature as indicated in Additional File 1: Table S1 with the 3D6 antibody, diluted in PBS containing 3% normal goat serum (Jackson ImmunoResearch Laboratories, 005–000-121), followed by incubation with anti-mouse Alexa Fluor 546 (Life Technologies, A10036) for 1 h at room temperature. After 4 washes alternating between PBS and PBST, sections were incubated for 10 min in 50% ethanol and PBS containing filtered 0.0015% Thioflavin S (Sigma-Aldrich, T1892). Sections were counterstained with NucRed Dead 647 ReadyProbes reagent (Thermo Fisher Scientific, R37113), coverslipped with ProLong Diamond Antifade Mountant (Thermo Fisher Scientific, P36970), and imaged with a BZ-X710 automated microscope system (Keyence) and an Aperio VERSA automated slide scanner (Leica Biosystems).

For immunostaining of other markers, sections were first quenched with 3% hydrogen peroxide and 10% methanol in PBS for 15 min to block endogenous peroxidase activity, followed by incubation for 1 h in 10% normal donkey serum, 1% non-fat dry milk, 0.2% gelatin, and PBS containing 0.5% Triton X-100. For c-Fos immunostaining, sections underwent antigen retrieval by incubating in citrate buffer (9.4 mM citric acid, 41 mM sodium citrate, pH 6) for 10 min at 100° C before the blocking step. Sections were then incubated as indicated in Additional File 1: Table S1 with antibodies against calbindin, neuropeptide Y (NPY), or c-Fos diluted in 3% normal donkey serum, 0.2% gelatin, and PBS containing 0.5% Triton X-100 overnight at 4° C. Sections were incubated in biotinylated anti-rabbit or anti-mouse secondary antibody. Signal was enhanced and visualized with an Elite ABC kit (Vector Laboratories) and 3,3′-diaminobenzidine (DAB) tetrahydrochloride kit (Vector Laboratories). Bright-field images were acquired with an Axio Scan.Z1 slide scanner (ZEISS) at 10x objective. Following previously described protocols [87], optical density measurements of calbindin and NPY immunoreactivity in dentate gyrus were measured using ImageJ software version 1.47 (http://imagej.nih.gov/ij) and c-Fos quantitations were performed by manual counting of positive cells in dentate gyrus by a researcher blinded to genotype.

To assess the activation state of microglia, sections were immunoperoxidase-stained with an Iba1 antibody using DAB for development. Images were acquired at 40x with an Aperio VERSA automated slide scanner (Leica Biosystems). The average number and length of microglial processes were quantified with the AnalyzeSkeleton (2D/3D) plugin of ImageJ as described [88]. The number and size of microglia were quantified as described [89] using a macro developed in-house. However, the macro was modified to replace AdaptiveThreshold with IsoData thresholding.

To visualize plaque-associated microglia in sections double-stained with Thioflavin S and anti-Iba1, the Iba1 labeling was enhanced with the Tyramide Signal Amplification (TSA) Plus Cyanine 5 kit (Akoya Biosciences). Images were acquired with an Olympus FV3000 Laser Scanning Confocal Microscope using 20x or 40x objectives and a 4x optical zoom.

### Electroencephalography (EEG) recordings and analysis

Lightweight EEG plugs were constructed in-house by soldering four Teflon-coated silver wire electrodes (0.125 mm diameter) to a multichannel electrical connector. After anesthetizing mice with isoflurane, EEG electrodes were surgically implanted under the skull and over the left and right frontal cortex (+1 mm anteroposterior (AP) and ±1 mm mediolateral (ML) relative to bregma) and the left and right parietal cortex (−2 mm AP and ±2 mm ML relative to bregma) as described [90–92]. The left frontal cortex was used as the reference electrode. Mice were allowed to recover from surgery for at least 2 weeks before EEG recordings began. Digital EEG activity and videos of their locomotor activity were recorded with a PowerLab data acquisition system. All signals were acquired at a sampling rate of 1000 Hz.

EEG recordings were analyzed with LabChart 7 Pro software (ADInstruments). Individual epileptiform spikes were detected automatically with a macro written in LabChart 7 as described in [90]. Briefly, deflections were identified as epileptiform spikes if their amplitude was ≥4-fold the average baseline of the trace and the absolute value of the second derivative of the slope was ≥10^4^. EEG traces and videos were also evaluated by an investigator blinded to genotype and treatment of the mice. Spikes judged to have resulted from movements or other artifacts were removed before quantitative analyses. A subset of spikes was classified as spike-and-wave discharges (SWDs) based on visual inspection. SWDs were defined as clusters of ≥4 spike-and-wave components occurring during ≤1 s. If a gap of ≥500 ms occurred between consecutive SWDs, they were considered separate events, whereas SWDs separated by <500 ms were considered a single event. The frequency of SWDs was quantified by counting the number of SWDs per hour. Calculations of spike frequencies (spikes/h) focused on individual spikes and excluded spikes that formed part of SWDs. Spectral analysis was performed by subjecting EEG segments to a fast Fourier transform (FFT) using a Hann cosine-bell window with no overlap between windows as described [90].

### Behavioral testing

Mice were group-housed between behavioral tests. The investigators were blinded to their genotype and treatment. Behavioral tests were administered during daytime and in the following order.

#### Elevated plus maze

The elevated plus maze (Kinder Scientific) consisted of two open arms (without walls) that intersected at 90° with two closed arms (with walls). The maze was positioned 63 cm above the ground. Movements were recorded by breaks of infrared beams positioned along the length of the open and closed arms. Before testing, mice were allowed to acclimate to the dimly-lit testing room for 1 h. During testing, mice were placed at the intersection of the open and closed arms and allowed to freely explore the maze for 10 min. The maze was cleaned with 70% ethanol between mice.

#### Open field

Spontaneous activity in an open field was assessed in a clear plastic chamber (41 × 41 × 30 cm) with photobeam arrays that automatically detected horizontal (16 × 16 photobeams) and vertical (16 photobeams) movements (Flex-Field/Open Field Photobeam Activity System, San Diego Instruments). Mice were allowed to acclimate to the testing room under normal light for 1 h before testing. During testing, mice were placed in the center of a clear plastic chamber and allowed to explore the chamber for 15 min. The chamber was cleaned with 70% alcohol between mice.

#### Morris water maze

The water maze consisted of a tank (122 cm in diameter) filled with water opacified with nontoxic white Tempera paint powder, and surrounded by large extramaze cues and room features. Mice were trained to locate a hidden platform (14 × 14 cm, submerged 1 cm below the water surface) in 2 learning sessions per day, with 2 trials administered in the morning session and 2 trials in the afternoon session (~15 min intertrial and ~3 h intersession intervals). The location of the hidden platform remained the same throughout spatial training but the drop locations on the side of the tank varied semi-randomly between trials. Each trial began by placing the mouse into the water facing the wall of the tank. Mice were then allowed to swim for a maximum of 60 s or until they located the platform. If the 60 s trial elapsed without locating the hidden platform, mice were gently guided to it by the researcher’s hand. Mice were required to remain on the platform for a minimum of 10 s before they were removed from the tank and returned to their home cage. Spatial memory for the location of the hidden platform was tested in a 60 s probe trial performed 24 h after the final training trial. For the probe trial, the drop location was the farthest location away from where the platform was located during spatial training. Swim paths, latencies and speed were recorded and analyzed with the EthoVision XT video tracking system (Noldus Information Technology).

#### Active place avoidance

The testing chamber consisted of a square rotating arena with a grid floor (81 × 81 cm; Bio-Signal Group Corp.). A smaller, clear plastic circular enclosure (40 cm in diameter) with a circular lid was placed on the center of the grid floor. The arena was surrounded by large distal spatial cues. A 60°  wedge within the 40 cm arena was designated as the aversive zone and was maintained in a constant position relative to the spatial cues outside the arena. One 10-min trial was performed per day over the course of 4 consecutive days. During the first day, a habituation trial was conducted in which the mice were placed inside the enclosure, the arena was rotated clockwise (1 RPM), and the mouse was allowed to freely explore for 10 min with the aversive zone stimulation deactivated. On days 2–4, mice were placed inside the enclosure and allowed to freely explore the rotating arena for 10 min with the stimulus generator activated for foot shock delivery upon entry into the aversive zone. A Tracker video tracking system (Bio-Signal Group Corp.) was used to determine when the mice were inside the aversive zone. Upon entry, a 0.2 mA shock was delivered for 500 ms and repeated every 1.5 s until the mice left the aversive zone wedge. The behavior of mice was video recorded and analyzed with Tracker (Bio-Signal Group Corp., Acton, MA). The arena was cleaned with 70% alcohol between mice.

### Statistical analysis

Statistical tests for each dataset are described in the figure legends. Statistical analysis was performed using Prism 7 or 8 (GraphPad Software) or the statistical programming language R (http://www.R-project.org/). Normality was assessed using the D’Agostino-Pearson omnibus test. Data that was not normally distributed was log transformed for statistical analysis if the transformation resulted in a normal distribution and allowed for analysis by parametric tests. Variance was assessed with the *F* test or Bartlett test for normally distributed data and with the Brown-Forsythe test for non-normally distributed data. For comparisons of two groups with normal distributions and equal variances, two-tailed unpaired *t* test was used. For comparisons of two groups with normal distributions but unequal variances, two-tailed unpaired *t* test with Welch correction was used. For comparison of three or more groups with normal distributions and equal variances, one-way or two-way analysis of variance (ANOVA) with Holm-Sidak post-hoc testing was used. For comparisons of multiple groups with normal distributions but unequal variances, multiple Welch t-test with Holm-Sidak correction were used. For comparison of multiple groups with non-normal distributions and equal variances, Kruskal-Wallis test with Dunn correction were used. For comparison of multiple groups with non-normal distributions and unequal variances, permutation tests with Holm-Sidak correction were used.

For analysis of learning performance in the Morris water maze (latency, distance, and cumulative search error) and active place avoidance (entrances to aversive zone and maximum time of avoidance), raw dependent measures were transformed into rank summary scores using the Excel PERCENTRANK function, which returns the rank of a value in a data set as a percentage of the data set. Rank summary scores were calculated for each mouse relative to the scores of all mice of all genotypes obtained in any given trial. One rank summary score was then calculated for each mouse by averaging all of its rank summary scores across all training trials. For hidden platform learning in the Morris water maze, trial 1 was excluded from the calculation of rank summary scores because it represents the very beginning of task acquisition [93]. Quadrant preference in the probe trial of the Morris water maze test was assessed by two-way ANOVA and Holm-Sidak correction.

Unless indicated otherwise, values reported are means ± standard error of the mean (SEM). In assays where absolute quantification was not performed, values were expressed relative to each other based on signals (e.g., counts or intensities) obtained in any given assay. When further data normalization was indicated, the level in a specified control group was arbitrarily defined as 1.0. Differences were considered significant at *P* < 0.05.

## Results

### Expression of APP and APP metabolites across lines

**Fig. 1:**
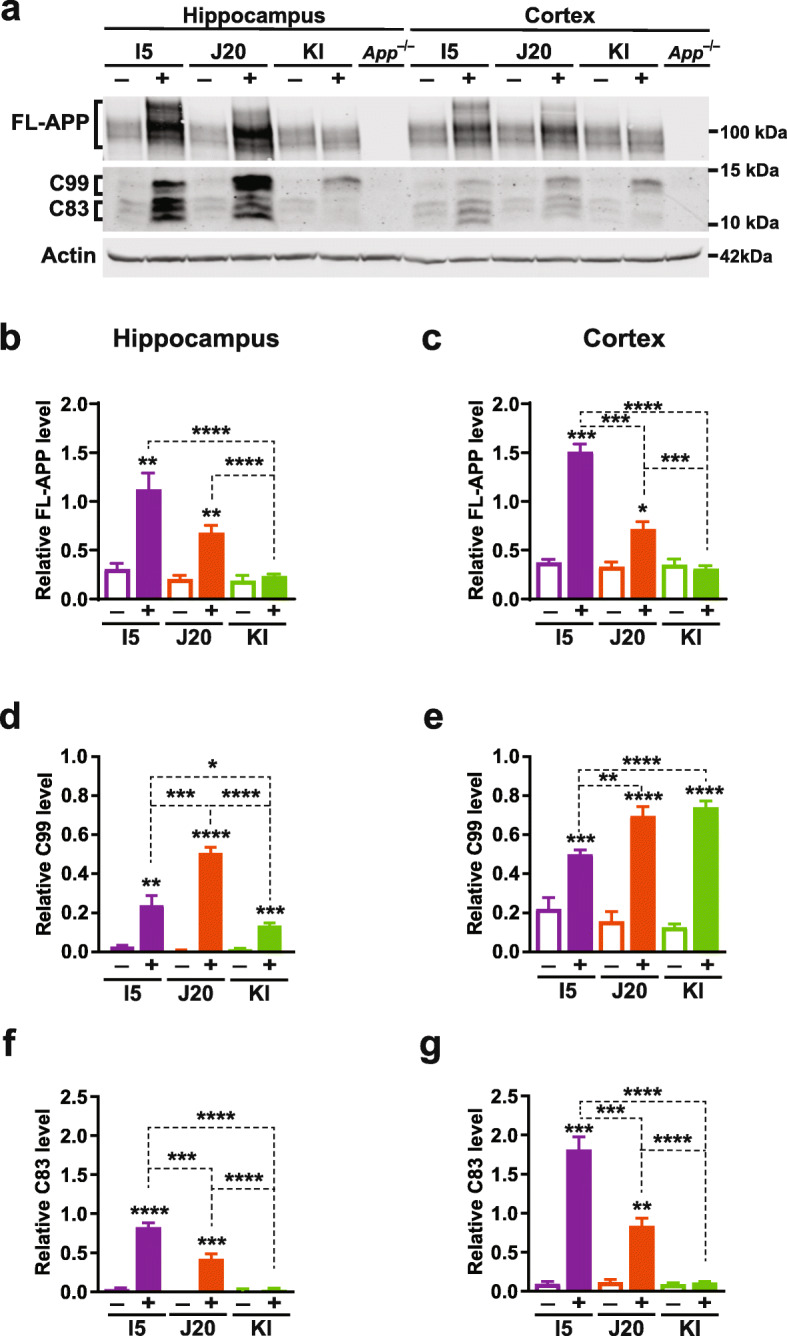
APP and APP C-terminal fragment levels. **a–g** Cortical (**a, c, e, g**) and hippocampal (**a, b, d, f**) levels of full-length (FL) APP and APP C-terminal fragments (CTFs) were determined in 6–8-month-old genetically modified mice (+) and WT controls (−) from the indicated lines by western blot analysis with antibodies 22C11 (epitope: amino acids 66–81 of the APP N-terminus) and CT15 (epitope: amino acids 680–695 of the APP C-terminus). **a** Western blot depicting bands representing APP holoprotein, C99 (also known as β-CTF), and C83. Actin-α1 was used as a loading control. **b–g** Quantitations of relative APP (**b, c**), C99 (**d, e**), and C83 (**f, g**) levels. The same set of J20 standards was included in each blot to normalize signals across blots as described in Materials and Methods. *n* = 5–15 mice per group. *P < 0.05, **P < 0.01, ***P < 0.01, **** *P* < 0.0001 vs. WT from same line or as indicated by brackets, based on multiple Welch t-test (**b, d–f**) or permutation test (**c, g**), with Holm-Sidak correction. Values are means ± SEM

We first compared hippocampal and cortical levels of APP and APP C-terminal fragments (CTFs) in I5, J20 and KI mice at 6–8 months of age. When we originally generated lines I5 and J20, they had comparable levels of APP overexpression [24]. By western blot analysis with an antibody that recognizes human and mouse APP, I5 mice now had 3–4-fold and J20 mice 2–3-fold higher overall APP levels than WT controls (Fig. 1a–c), suggesting that transgene expression levels diverged in these lines during many years of breeding, possibly due to selection pressures imposed by the FAD mutations in J20 mice or random factors. As expected [56], APP levels in the hippocampus and cortex of KI mice were similar to those in WT controls (Fig. 1a–c). In I5 and J20 mice, but not in KI mice, APP levels were higher in the hippocampus than the cortex (Additional File 1: Figure S1a).

Compared with WT controls, all three groups of genetically modified mice had marked elevations of C99 fragments (Fig. 1a, d, e), including KI mice, whose cortical C99 levels were as high as those of J20 mice (Fig. 1a, e). In contrast, C83 levels were elevated in I5 and J20 mice, but not in KI mice (Fig. 1a, f, g). Furthermore, J20 mice had higher C99 levels but lower C83 levels than I5 mice (Fig. 1a, d–g). These results likely reflect overexpression of hAPP (I5 and J20 mice), effects of the Swedish (J20 and KI mice) and Arctic (KI mice) mutations [5, 6, 24, 94–97], and differences in the expression patterns directed by the human PDGF-β promoter (I5 and J20 mice) versus endogenous *App* regulatory sequences (KI mice).

**Fig. 2:**
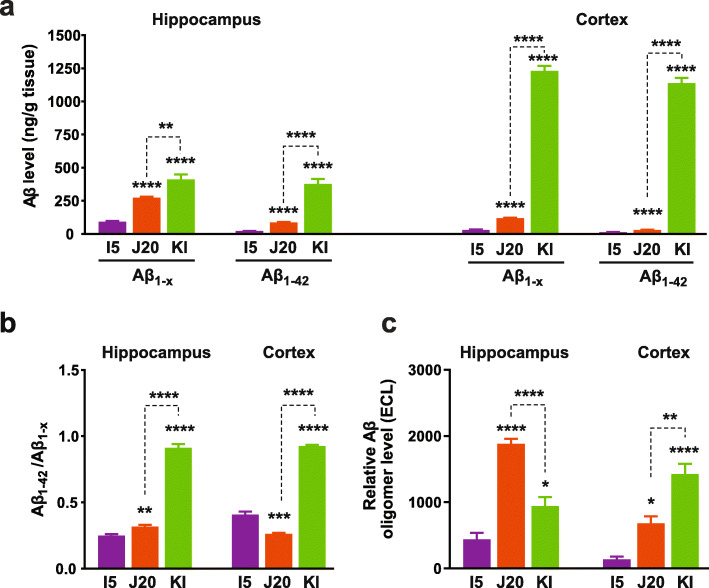
Aβ and Aβ oligomer levels. **a**, **b** Overall levels of Aβ_1-x_ and Aβ_1–42_ (**a**) and Aβ_1–42_/Aβ_1-x_ ratios (**b**) in the hippocampus and cortex were determined by ELISA in 2–3-month-old mice of the indicated genotypes. (**c**) Relative hippocampal and cortical Aβ oligomer levels were determined in PBST fractions from 2 to 3-month-old I5, J20 and KI mice by 3D6/3D6 MSD ELISA. Based on a comparison of WT and *App*^−/−^ mice (Additional File 1: Fig. S4e), the average electrochemiluminescence (ECL) signal in WT controls was considered background and subtracted from ECL signals obtained in individual genetically modified mice. *n* = 5–12 mice per group. *P < 0.05, **P < 0.01, **** *P* < 0.0001 vs. I5 or as indicated by brackets, based on multiple Welch t-tests with Holm-Sidak correction (**a, b**) or one-way ANOVA and Holm-Sidak correction (**c**). Values are means ± SEM

**Fig. 3:**
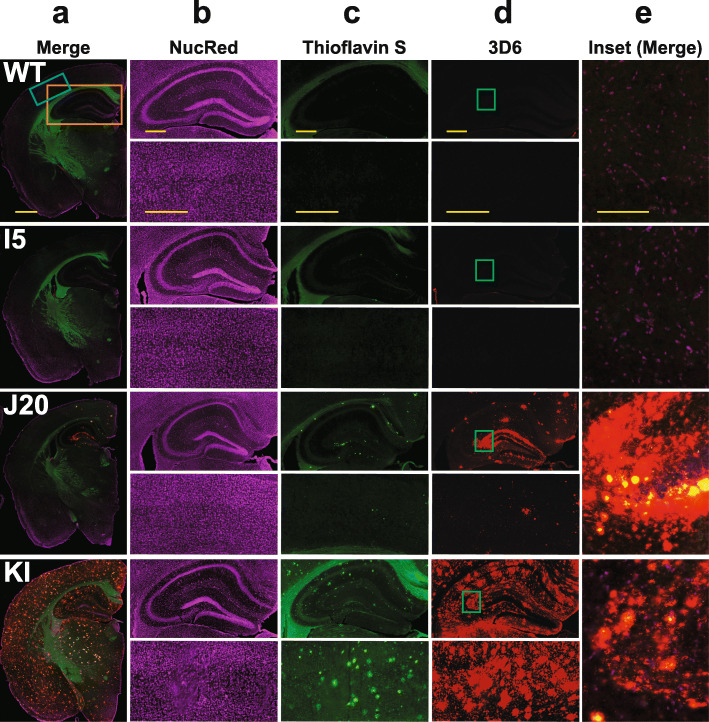
Amyloid deposition. **a–e** Coronal brain sections from 8 to 9-month-old mice of the indicated genotypes were labeled with nuclear red (NucRed) and stained for Aβ deposits with Thioflavin S or the 3D6 antibody. Columns (**a**) and (**e**) show merges of all three stains. Column (**a**) depicts hemibrains (scale bar: 1.2 mm), columns (**b–d**) show hippocampus (top) and neocortex (bottom) (scale bars: 600 μm), and column (**e**) provides a magnified view (scale bar: 150 μm) of the inset in column (**d**)

These variables also affected the levels (Fig. 2) and deposition (Fig. 3) of Aβ. The 3D6 and 266 antibodies recognize amino acids 1–5 and 13–28 of Aβ, respectively (Additional File 1: Table S1), and, thus, detect Aβ_1–40_ and Aβ_1–42_ as well as C-terminally truncated or extended Aβ species [98], collectively referred to here as Aβ_1–x_. To measure Aβ_1–42_, we used the 21F12 antibody, which specifically recognizes the C-terminal 10 amino acids of this peptide (Additional File 1: Table S1), in combination with 3D6 [73]. Fibrillar and non-fibrillar Aβ deposits were detected by 3D6 immunostaining, whereas fibrillar Aβ deposits containing β-pleated sheet structures were detected by Thioflavin S staining.

While I5 mice had the highest APP levels (Fig. 1a–c), they showed only minimal elevations in Aβ levels at 2–3 months (Fig. 2a) and no evidence for Aβ deposition (Fig. 3) at 9–10 months of age, consistent with previous observations [24, 51]. By 9–10 months of age, Aβ deposition was more widespread in KI than J20 mice (Fig. 3). KI mice showed prominent Aβ deposition in cortical and subcortical regions, including in the striatum and thalamus, which can also be areas of prominent Aβ deposition in humans with autosomal dominant AD [99, 100]. J20 mice showed prominent Aβ deposition in the hippocampus and also some cortical Aβ deposits at this age, but no Aβ deposition in subcortical structures (Fig. 3). Possibly due to effects of the Arctic mutation [6, 94, 97], Aβ deposition also occurs earlier in KI than J20 mice (refs. [24, 56] and data not shown), which may have contributed to the higher Aβ_1–42_ and Aβ_1–x_ levels and higher Aβ_1–42_/Aβ_1–x_ ratios in KI mice at 2–3 months of age (Fig. 2a, b). Because *App*^NL-F^ knock-in mice, which lack the Arctic mutation [56], have much lower hippocampal Aβ levels than J20 and *App*^NL-G-F^ (KI) mice (Additional File 1: Figure S1b and Fig. 2a), we decided to use KI mice for the current study.

To compare Aβ oligomer levels across I5, J20 and KI mice, we extensively validated (Additional File 1: Figures S2–5) and used the ELISA-based approach Yang and colleagues developed [47]. It employs the same anti-Aβ antibody for capture and detection (Additional File 1: Figure S2a, b) and will be referred to as 3D6/3D6 MSD ELISA. Cortical levels of Aβ oligomers at 2–3 months were higher in KI than J20 mice (Fig. 2c), as one might have expected from the higher cortical levels of Aβ levels and Aβ_1–42_/Aβ_1–x_ ratios in KI mice (Fig. 2a and b). However, hippocampal levels of Aβ oligomers at 2–3 months were actually higher in J20 than KI mice (Fig. 2c), even though hippocampal Aβ levels and Aβ_1–42_/Aβ_1–x_ ratios at this age and the extent of hippocampal Aβ deposition at 9–10 months were lower in J20 than KI mice (Figs. 2a, b and 3).

### Non-convulsive epileptiform activity detected in all three lines

**Fig. 4:**
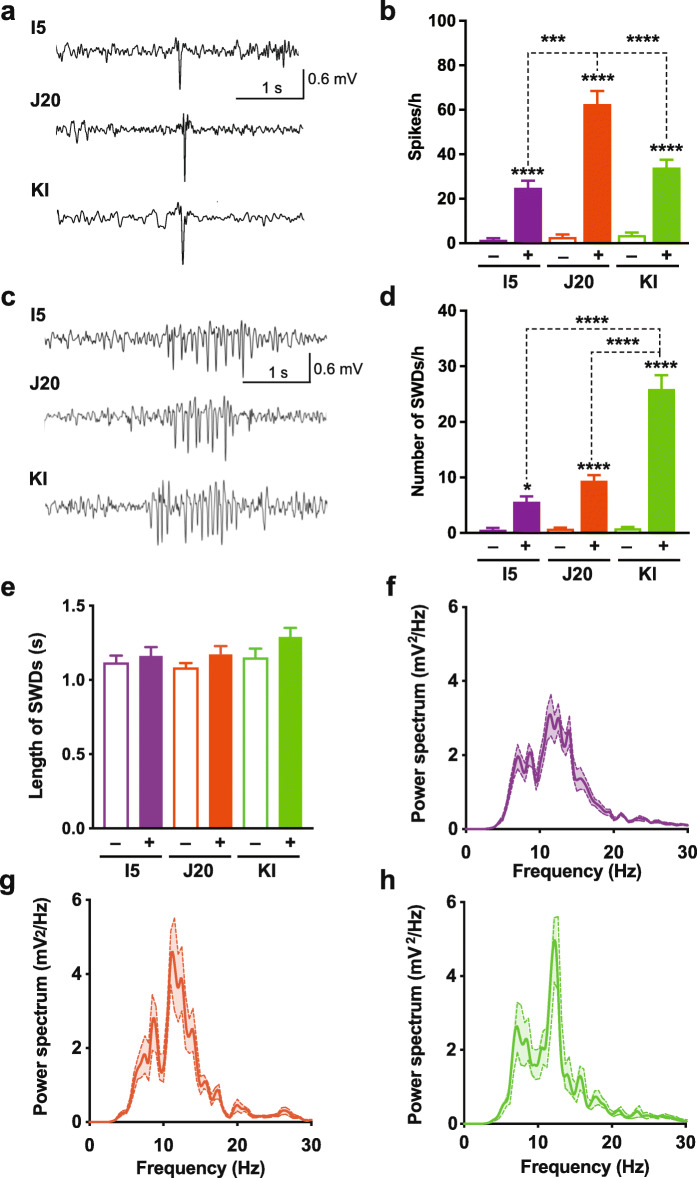
Non-convulsive epileptiform activity. Intracranial EEG recordings were obtained in the resting state from 8 to 12-month-old genetically modified mice (+) and WT controls (−). **a** Traces depict typical epileptiform spikes. **b** Spike frequencies measured during a 12-h period. **c** Representative SWDs. **d, e** Frequency (**d**) and length (**e**) of SWDs measured during a 12-h period. *n* = 10–12 mice per group. **f–h** Power spectral density analysis (*n* = 5 mice per group). Two waveforms were analyzed per genetically modified mouse. Note the spectral peak at 12 Hz. **P < 0.01, ***P < 0.001, ****P < 0.0001 vs. WT from same line or as indicated by brackets, based on one-way ANOVA followed by Holm-Sidak test. Values are means ± SEM

Intracranial EEG monitoring revealed abnormal increases in epileptiform activity in I5, J20 and KI mice that was unaccompanied by myoclonic jerks or other abnormal movements, as compared to WT controls (Fig. 4a, b). We did not observe convulsive seizures in any of the mice during this study (data not shown). Although APP levels were higher in I5 than J20 mice (Fig. 1a–c), I5 mice had lower spike frequencies than J20 mice (Fig. 4b). And despite the absence of APP overexpression in KI mice (Fig. 1a–c), these mice also had more epileptiform spikes than WT controls, although their spike frequencies were lower than those of J20 mice (Fig. 4b). A previous comparison of J20 mice with *App*^NL-F^ knock-in mice detected epileptiform activity in the former but not the latter model [101], but this difference is difficult to interpret because of the much lower Aβ levels in the *App*^NL-F^ knock-in mice (Additional File 1: Figure S1b).

Because of the prominent deposition of Aβ in the thalamus of KI mice (Fig. 3), we also assessed spike-and-wave discharges (SWDs), which often arise from alterations in thalamo-cortical circuits [102–105]. Although SWDs were observed in all three lines of mice, they were most prominent in KI mice (Fig. 4c, d). Across lines, the length (i.e., duration) of SWD events was similar (Fig. 4e) and the power spectral density of SWDs peaked around 12 Hz (Fig. 4f–h). SWDs in KI mice were associated with synchronized burst firing in the somatosensory ventrobasal thalamic complex (not shown). These electrophysiological features of SWDs are similar to those observed by others in hAPP mice [64, 106, 107] and in mouse and rat models of genetic absence-type epilepsy [105, 108–110] or brain injury-induced epilepsy [111].

**Fig. 5:**
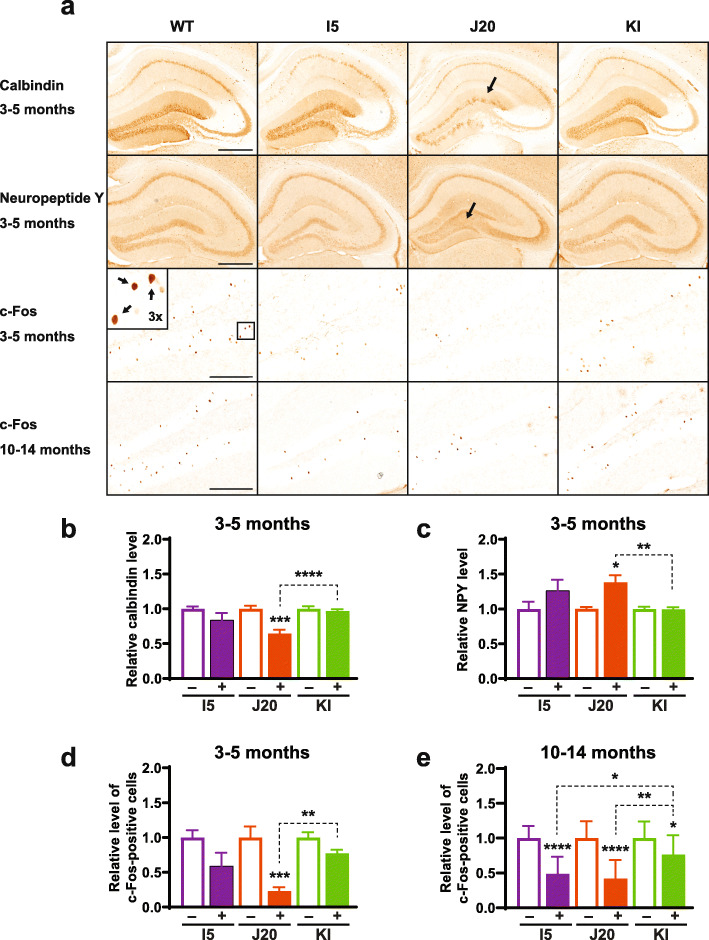
Hippocampal indicators of epileptiform activity. **a–e** Coronal brain sections were obtained from genetically modified mice (+) and WT controls (−) at 3–5 (**a–d**) or 10–14 (**a, e**) months of age and immunostained for calbindin (**a, b**), neuropeptide Y (NPY) (**a, c**), or c-Fos (**a, d, e**). **a** Photomicrographs depicting typical levels and distributions of calbindin, NPY and c-Fos immunoreactivities in the hippocampus and dentate gyrus from mice of the indicated genotypes and ages. Arrows indicate depletion of calbindin in the molecular layer of the dentate gyrus (top) and increased NPY labeling of mossy fibers (middle) of a J20 mouse, and a 3-fold magnified inset image of c-Fos-positive granule cells (bottom) representing the boxed area in the dentate gyrus of a WT control. Scale bars: 500 μm (top and middle row), 200 μm (bottom row). **b–e** Quantitation of calbindin levels in the molecular layer of the dentate gyrus (**b**), NPY levels in the hilus of the dentate gyrus (**c**), and c-Fos-positive cells in the granular layer of the dentate gyrus (**d, e**). For each antigen, levels in genetically modified mice were expressed relative to mean levels in WT controls from the same line, which were defined as 1.0. *n* = 7–15 mice per group. *P < 0.05, **P < 0.01, ***P < 0.001, ****P < 0.0001 vs. WT from same line or as indicated by brackets, based on multiple Welch *t*-tests with Holm-Sidak correction (**b, c**), Kruskal-Wallis test with Dunn correction (**d**), or one-way ANOVA with Holm-Sidak correction (**e**). Values are means ± SEM

Epileptic activity that emanates from the cortex and spreads into the hippocampus is well known to trigger diverse adaptations in the latter structure. Even non-convulsive epileptiform activity can cause robust molecular alterations in the dentate gyrus, including reductions in calbindin and c-Fos levels in granule cells and ectopic expression of neuropeptide Y (NPY) in their mossy fiber axons [52, 53, 87, 112]. At 3–5 months of age, J20 mice displayed all three abnormalities, whereas I5 and KI mice showed only trends toward one or more of these alterations (Fig. 5a–d). By 10–14 months, I5 and KI mice also showed significant alterations in such epilepsy-related outcome measures (Fig. 5a and e).

Thus, the ability of APP to promote epileptiform activity is enhanced by overexpression and by the introduction of FAD mutations, but it does not have a simple relation to cortical or hippocampal levels of APP, C99, C83, Aβ_1–x_, Aβ_1–42_, Aβ_1–42_/Aβ_1–x_ ratios, Aβ oligomers, or Aβ deposits.

### Behavioral abnormalities observed in all three lines

**Fig. 6:**
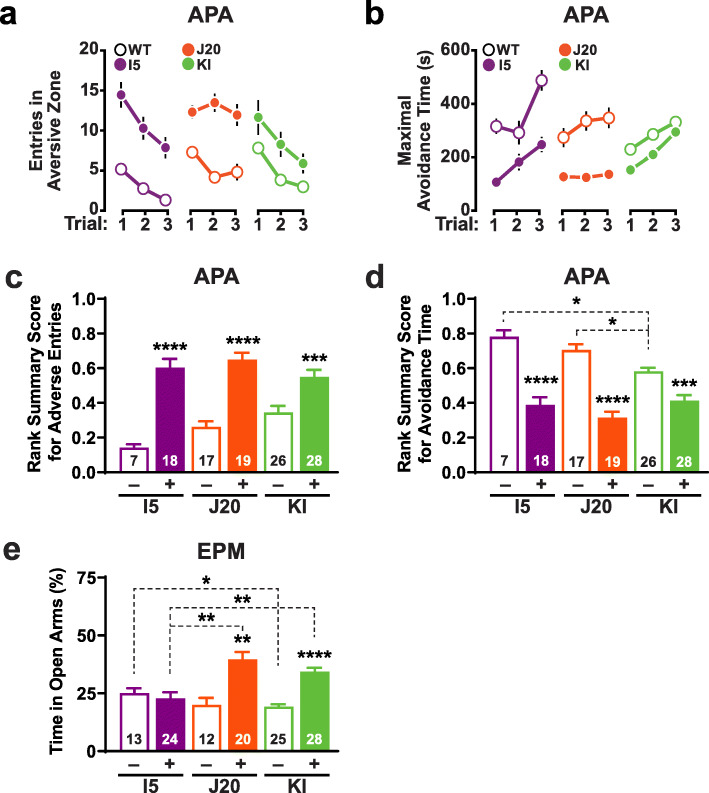
Behavioral alterations. **a–d** Spatial learning and memory of genetically modified mice (+) and WT controls (−) from each line were assessed at 6.0–7.5 (I5 and J20) or 8–11 (KI) months of age in the active place avoidance (APA) test. The number of mice analyzed per group is indicated in the bar graphs. **a, b** Learning behavior was reflected in decreasing entries into the aversive zone (**a**) and increasing maximal avoidance time of that zone (**b**). **c, d** These measures were quantified by calculating rank-summary scores as described in Methods. Impaired performance is reflected in higher scores in (**c**) and lower scores in (**d**). **e** Genetically modified mice (+) and WT controls (−) from each line were assessed in the elevated plus maze (EPM) at 4–5 (I5 and J20) or 6–9 (KI) months of age. The percentage of time they spent in the open arms of the maze is shown. *P < 0.05, ***P < 0.001, ****P < 0.0001 vs. WT from same line or as indicated by brackets, based on one-way ANOVA and Holm-Sidak correction (**c, d**) or multiple Welch t-tests with Holm-Sidak correction (**e**). Some error bars in (**a, b**) are not visible because they are very small. Values are means ± SEM

To evaluate spatial learning and memory, we tested mice at 6–11 months of age in an active place avoidance paradigm [113]. Mice were tested approximately 2 months after spatial learning and memory deficits were reported to become detectable in the respective lines [56, 114]. I5, J20 and KI mice all displayed deficits in this task relative to age- and line-matched WT controls (Fig. 6a–d). These findings are broadly consistent with previous results obtained in these or similar models [60, 115–119].

Both J20 and KI mice spent significantly more time on the open arms of an elevated plus maze than WT controls, whereas I5 mice did not (Fig. 6e), raising the possibility that this abnormality depends on Aβ or C99 levels rather than APP levels. Although this behavioral phenotype can signify reduced anxiety [120], it is also observed after entorhinal cortex lesions [121] and has been variably interpreted as impaired learning of an aversive environment [122] or disinhibition [123].

### BACE1 inhibition does not reduce functional abnormalities in J20 mice

**Fig. 7:**
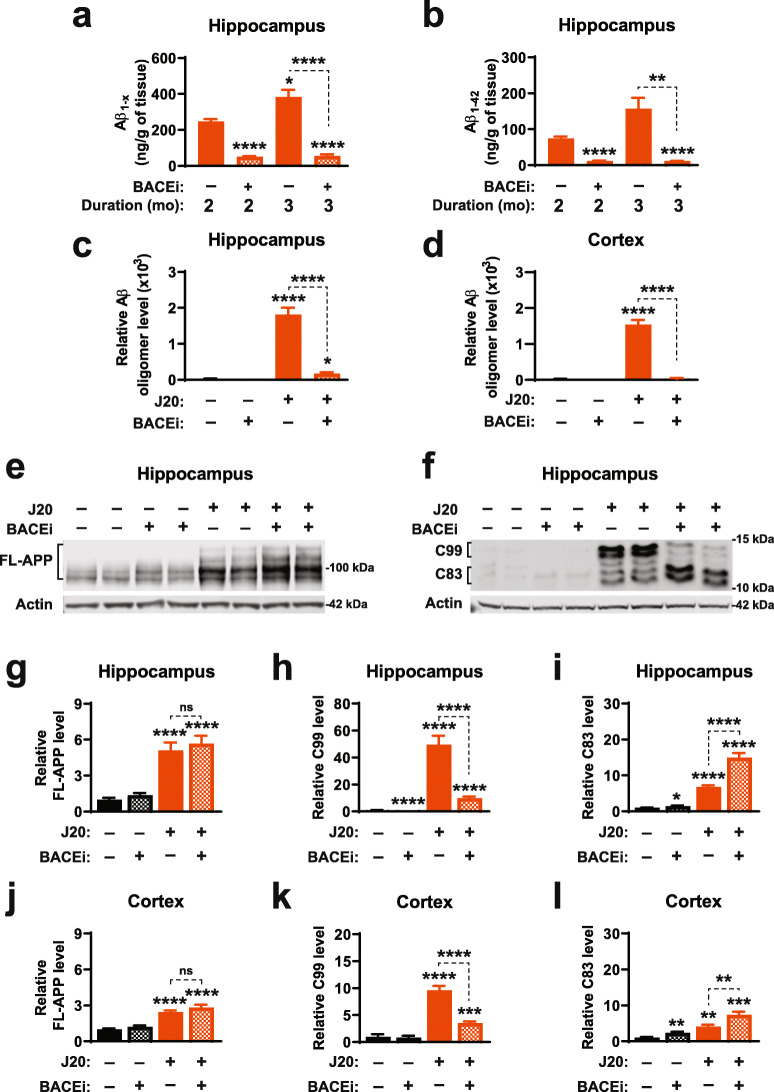
BACEi-induced alterations in APP metabolism. **a–l** Beginning at 1 month of age, J20 mice (+) and WT (−) controls were treated for 2 or 3 months (**a, b**) or 6–8 months (**c–l**) with the BACEi NB-360 (+) or placebo (−). Levels of APP and its metabolites were measured in the indicated brain regions at the end of treatment. **a, b** Hippocampal levels of Aβ_1-x_ (**a**) and Aβ_1–42_ (**b**) were determined by ELISA. **c, d** Levels of Aβ oligomers in the hippocampus (**c**) and cortex (**d**) were determined by 3D6/3D6 MSD ELISA as in Fig. 2c. Average signals in placebo-treated WT mice were used for background subtraction. **e, f** Western blots depicting hippocampal signals for full-length (FL)-APP (**e**) and CTFs (**f**). Actin was used as a loading control. **g–l** Quantitations of western blot signals for the indicated brain regions corresponding to FL-APP (**g, j**), C99 (**h, k**) and C83 (**i, l**). Mean levels in placebo-treated WT mice were defined as 1.0. *n* = 4–6 (**a, b**), 7–14 (**c, d**), and 8–16 (**g–l**) mice per group. All groups in (**a, b**) included males and females, while groups in (**c–l**) consisted of females only, as male mice were used for the behavioral analyses in Fig. 8. *P < 0.05, **P < 0.01, ***P < 0.001, ****P < 0.0001 vs. placebo-treated WT or as indicated by brackets, based on multiple Welch t-tests with Holm-Sidak correction. Values are means ± SEM

To further explore the relationship between functional abnormalities and APP metabolites, we treated J20 mice with a BACE1 inhibitor (NB-360) [71, 72]. We selected the J20 line for this experiment because it consistently shows robust levels of network dysfunction and behavioral deficits. Starting at 1 month of age, J20 mice and WT controls were placed on bacon-flavored chow that contained NB-360 (BACEi, 500 mg/kg of chow) or on bacon-flavored chow without the drug (placebo) for 8 or 12 weeks. The BACEi treatment resulted in an estimated average oral NB-360 intake of ~80 mg/kg body weight/day and markedly reduced hippocampal levels of Aβ_1–x_ and Aβ_1–42_ in J20 mice (Fig. 7a, b). It also effectively reduced Aβ oligomer levels in their hippocampus and cortex (Fig. 7c, d). In addition, BACEi treatment decreased C99 levels and increased C83 levels in the hippocampus and cortex of J20 mice (Fig. 7e–l), providing further evidence for proper target engagement in the brain.

**Fig. 8:**
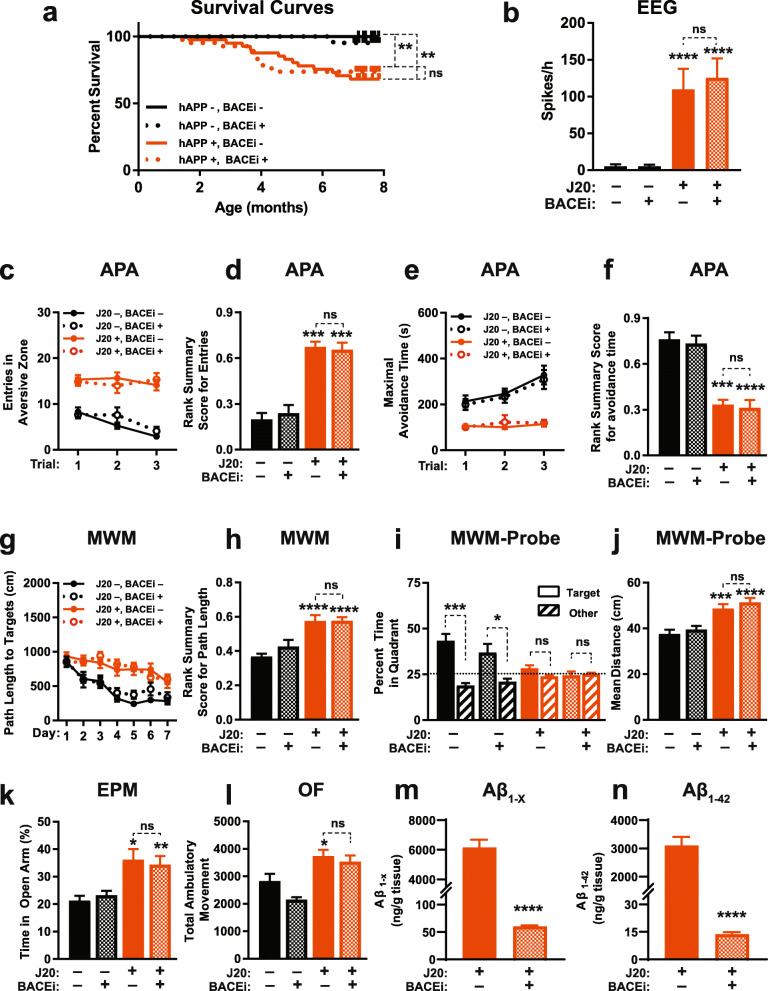
Early BACEi treatment does not prevent functional abnormalities in J20 mice. **a–l** J20 mice (+) and WT (−) controls were treated with the BACEi NB-360 (+) or placebo (−) starting at 1 month of age and continuing throughout the observation period (**a**), behavioral testing at 4–6 months (**c–l**), EEG recordings at 6–7 months (**b**), and hippocampal Aβ measurements at 7–8 months (**m, n**) of age. **a** Survival curves. **P < 0.01 or not significant (ns) as indicated by brackets, based on Mantel-Cox log-rank test. The trend toward an acceleration in mortality in BACEi-treated J20 mice around 4 months was not statistically significant (*P* = 0.07, based on log-rank test applied to data of mice from either J20 group that survived at least 4 months). **b** Epileptiform spike frequencies determined in resting mice during 8-h recording periods. **c–f** Learning and memory in the active place avoidance (APA) test was quantified as in Fig. 6a–d. **g, h** Learning behavior in the Morris water maze (MWM) test was reflected by reductions in the path length that mice required to locate a hidden platform (**g**) and was quantitated by a rank summary score (**h**), which was calculated as described in Methods. **i, j** Spatial learning and memory retention were assessed in a probe trial 24 h after the last training trial, in which we determined whether mice favored the quadrant in which the platform was previously located (**i**) and their mean distance from the original target location averaged over each second of the probe trial (**j**). The target preference index was calculated by dividing the percentage of time mice spent in the target quadrant by the percentage of time they spent in non-target quadrants. **k** Percentage of time mice spent in the open arms of an elevated plus maze (EPM). **l** Locomotor activity in the open field (OF) was recorded for 15 min. (**m, n**) Hippocampal levels of Aβ_1-x_ (**m**) and Aβ_1–42_ (**n**) were determined by ELISA. Three behaviorally tested J20 mice (2 BACEi-treated and 1 placebo-treated) were not included in this analysis because they died after the behavioral testing and before Aβ measurements could be carried out. *n* = 21–42 (**a**), 11–16 (**b**), 11–17 (**c–f**), 12–17 (**g–l**), and 14–15 (**m, n**) mice per group. All groups in (**a**) included males and females, whereas groups in (**b–n**) consisted of males only to reduce variability in behavioral performance. *P < 0.05, **P < 0.01, ***P < 0.001, ****P < 0.0001 vs. placebo-treated WT or as indicated by brackets, based on two-way ANOVA with Holm-Sidak correction (**b, h, i, j**), Kruskal-Wallis test with Dunn correction (**d, f, l**), multiple Welch t-tests with Holm-Sidak correction (**k**), or two-tailed unpaired *t* test with (**m**) or without (**n**) Welch’s correction. Values are means ± SEM

Despite these robust effects on APP metabolism, BACEi treatment did not reduce a range of functional abnormalities in J20 mice, including premature mortality (Fig. 8a), epileptiform activity (Fig. 8b), deficits in the active place avoidance (Fig. 8c–f) and Morris water maze (Fig. 8g–j) tests, reduced avoidance of open arms in the elevated plus maze (Fig. 8k) and locomotor hyperactivity (Fig. 8l). J20 mice and WT controls treated with BACEi or placebo showed comparable swim speeds in the cued component of the Morris water maze test (Additional File 1: Figure S6).

Measurements of hippocampal Aβ levels after the behavioral testing revealed markedly reduced levels of Aβ_1-x_ and Aβ_1–42_ in BACEi-treated J20 mice (Fig. 8m, n), confirming that the BACEi treatment effectively inhibited amyloidogenic hAPP processing in this cohort of mice. Consistent with these results, BACEi treatment prevented the development of thioflavin S-positive amyloid plaques and of plaque-associated microgliosis in J20 mice (Additional File 1: Figure S7).

### BACE1 inhibition does not ameliorate sodium channel depletions in J20 mice

**Fig. 9:**
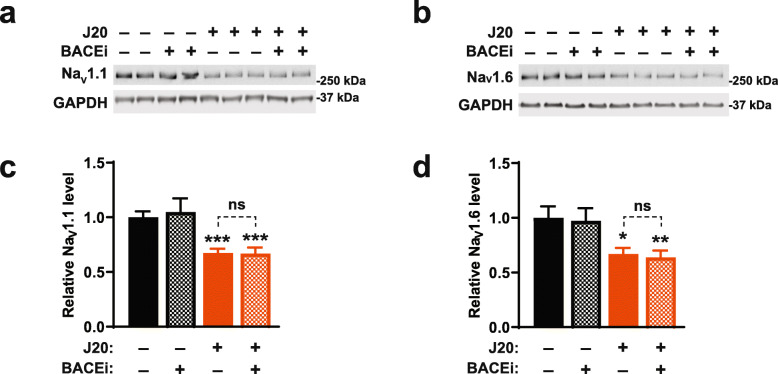
Early BACEi treatment does not prevent Na_V_1.1 depletion in J20 mice. **a–d** J20 mice (+) and WT controls (−) were treated with the BACEi NB-360 (+) or placebo (−) starting at 1 month of age. Na_V_1.1 and Na_V_1.6 levels in the parietal cortex were determined by western blot analysis at 7–9 months of age. **a, b** Images of western blots depicting signals for Na_V_1.1 (**a**) and Na_V_1.6 (**b**). GAPDH served as a loading control. **c, d** Quantitation of relative Na_V_1.1 (**c**) and Na_V_1.6 (**d**) levels. Average levels in placebo-treated WT mice were arbitrarily defined as 1.0. *n* = 8–16 female mice per group. *P < 0.05, **P < 0.01, ***P < 0.001, ****P < 0.0001 vs. placebo-treated WT mice or as indicated by brackets, based on multiple Welch t-tests (**c**) or one-way ANOVA (**d**) with Holm-Sidak correction. Values are means ± SEM

Evidence has been accumulating that multiple functional abnormalities in J20 mice, and possibly also some of those observed in humans with AD, are caused by impairments of inhibitory interneurons, which—in turn—result, at least in part, from the depletion of specific voltage-gated sodium channels [54, 60, 124]. Although the mechanisms underlying this depletion remain to be determined, BACE1-mediated cleavage of Na_V_β2 has been implicated in the hypofunction of AD-relevant sodium channels in APP transgenic mice [125, 126]. We examined sodium channel levels in the parietal cortex because we previously detected sodium channel depletions only in this brain region but not in the hippocampus [54]. In our study, BACEi treatment did not ameliorate the reductions of Na_V_1.1 and Na_V_1.6 levels in the parietal cortex of J20 mice (Fig. 9), providing a plausible explanation for the lack of functional improvements after BACEi treatment in this model and, possibly, also in humans with AD.

Because the hAPP minigene with which we generated the J20 line [24, 127] integrated into noncoding sequence of the *Zbtb20* gene [128], we compared Zbtb20 and Na_V_1.1 levels in the parietal cortex of hemizygous transgenic mice from line J20 with those in hemizygous *Zbtb20* knockout (*Zbtb20*^+/−^) mice [129] and WT controls. At 2 months of age, J20 mice already had significant reductions of Na_V_1.1, but not of Zbtb20, whereas the opposite was true for *Zbtb20* knockout mice (Additional File 1: Figure S8). It is therefore unlikely that the inability of BACEi to prevent sodium channel depletions and related functional deficits in J20 mice was due to alterations in Zbtb20 expression in this line.

## Discussion

The results of our study indicate that FAD-linked APP mutations can cause non-convulsive epileptiform activity, related immunohistochemical alterations, and cognitive deficits in the absence of APP overexpression, and that overexpression of WT hAPP can cause similar dysfunctions in the absence of such mutations. FAD mutations and APP overexpression appear to have synergistic effects, causing enhanced neural network dysfunction and premature mortality.

Many of our findings are consistent with observations in humans, in whom AD-like cognitive decline and epileptiform activity can result not only from the inheritance of FAD-linked APP mutations, but also from overexpression of WT hAPP resulting from duplications of the *APP* gene [60, 130] and from trisomy 21 [131], in which an additional WT *APP* allele is expressed from the extra copy of chromosome 21. Intriguing mechanisms have been identified by which WT hAPP may be overexpressed at synapses [132] and mutant forms of hAPP may accumulate in individual neurons also in sporadic AD [133]. Notably, non-convulsive epileptiform activity has been identified in over 40% of patients with sporadic AD [63].

The inability of BACEi treatment to reduce functional abnormalities in J20 mice also seems consistent with the negative outcome of clinical trials of other BACE1 inhibitors in humans [33, 34]. Why other groups observed beneficial effects of such drugs in earlier clinical trials [134] and in APP transgenic mice [13, 135–137] is unclear but may relate to differences in patient populations, mouse models or methods used.

At first glance, our comparative analysis of I5 and J20 mice would seem consistent with the notion that functional abnormalities in APP transgenic mice may result from the overexpression of APP rather than the pathological accumulation of Aβ [23, 70, 118, 138–142], except for aberrant behavior in the elevated plus maze. This abnormality was found in J20 but not I5 mice and, thus, may be more specifically related to the elevated levels of Aβ and C99 in J20 mice or to other consequences of the FAD mutations expressed in this model.

Notably, KI mice, which do not overexpress APP but have human FAD mutations and a humanized Aβ sequence in their mouse *App* alleles [56], displayed broadly similar functional abnormalities as J20 mice, including non-convulsive epileptiform activity, deficits in learning and memory, and aberrant behavior in the elevated plus maze. J20 and KI mice share the expression of FAD-mutant APP, albeit at different levels of production, as well as the abnormal accumulation of Aβ and C99, features they also have in common with FAD patients [143]. It is therefore impossible in such models and in the human condition to differentiate among the pathogenic contributions of these factors without additional manipulations. The same caveat applies to transgenic mice with regulatable hAPP expression [23]. Treatment with a γ-secretase inhibitor reduced behavioral deficits [138] but not epileptiform activity [70] in such models. Because the latter study did not quantify Aβ oligomer levels and these assemblies are cleared more slowly from the brain than other forms of Aβ after inhibition of γ-secretase [144], the inability of the γ-secretase inhibitor to reduce epileptiform activity may have resulted from an inadequate reduction of Aβ oligomer levels; however, C99 accumulation and unchanged levels of APP overexpression may have contributed as well. Born and colleagues did not detect epileptiform activity in *App*/*Psen1* double knockin mice [70], but it is unclear how Aβ and C99 levels in this model compare to those in J20 and KI mice.

Our finding that hippocampal Aβ oligomer levels at 2–3 months were higher in J20 than KI mice, even though hippocampal Aβ monomer levels and Aβ_1–42_/Aβ_1–x_ ratios at this age and the extent of hippocampal Aβ deposition at 9–10 months were lower in J20 than KI mice suggests that Aβ oligomer levels do not only depend on levels of Aβ_1–x_ and Aβ_1–42_ or on Aβ_1–42_/Aβ_1–x_ ratios, but also on other factors that can differ among brain regions such as local concentrations of pathological molecular chaperones [145–147].

Despite marked differences in the levels of specific APP metabolites among the three lines tested, all models developed epileptiform activity and behavioral deficits. Differences in the onset of these functional deficits across lines could be influenced by many variables, including promoter-dependent differences in the level and cell-specific distribution of APP expression as well as differences in the relative preponderance of APP isoforms expressed.

To differentiate the effects of Aβ oligomers and C99 from other effects of FAD-mutant APP that may lead to functional abnormalities, we treated J20 mice with a BACE inhibitor. Although this treatment effectively reduced brain levels of Aβ, Aβ oligomers and C99, it did not reduce epileptiform activity or deficits in learning and memory. Taken together, these results cast some doubt on the pathogenic importance of Aβ, Aβ oligomers and C99, and support the notion that APP and FAD-causing APP mutations contribute to the pathogenesis of AD through alternative mechanisms.

It is also worth considering Aη-α fragments in this context, which are derived from APP by η- and α-secretase cleavage and have been shown to impair synaptic and neuronal functions in acute hippocampal slices [148, 149]. However, while inhibition of BACE1, which cleaves η-CTFs, leads to a buildup of Aη-α fragments [148, 149], we did not observe any deficits in WT controls or worsening of functional abnormalities in J20 mice after BACEi treatment.

Because the J20 model shares many features with AD patients [60], in which BACE1 inhibitors also failed to prevent or slow cognitive decline [33, 34], the inability of BACEi to reduce functional abnormalities in J20 mice could be interpreted as high predictive value of the model. Since we started to treat J20 mice at 4 weeks of age, when they show no or only minimal cognitive deficits and no evidence for amyloid deposition [24, 94], our findings may also raise concerns about the future success of preventive clinical trials of BACE1 inhibitors in human FAD mutation carriers [134, 150, 151]. However, it would be incautious to speculate about the potential outcome of clinical trials based on results obtained in just one experimental model.

It is also important to consider additional factors that might have made functional abnormalities in J20 mice resistant to the prominent BACEi-mediated reductions in Aβ, Aβ oligomer and C99 levels. Transgene insertion-mediated mutagenesis deserves some discussion in this context. The hAPP minigene we used to generate the J20 line [24, 127] randomly integrated into noncoding sequence of the *Zbtb20* gene [128]. A previous western blot analysis [128] and the current study suggest that this insertional event did not significantly reduce Zbtb20 protein levels in AD-relevant brain regions of J20 mice, although J20 showed a trend toward Zbtb20 reduction in the parietal cortex that did not reach statistical significance. Most importantly, we found no reduction of Na_V_1.1 levels in the parietal cortex of hemizygous *Zbtb20* knockout mice, whereas this alteration is present in J20 mice as well as in humans with AD, and clearly contributes to functional impairments in J20 mice [54, 124]. We therefore consider it unlikely that alterations in Zbtb20 protein levels contributed to the inability of BACEi to ameliorate sodium channel depletions and related functional deficits in J20 mice.

Another and, in our view, more likely explanation of why BACE1 inhibition failed to improve functional abnormalities in J20 mice is the inability of the BACEi to prevent or reverse their Na_V_1.1 depletion. This molecular alteration strongly contributes to the behavioral and electrophysiological phenotype of the J20 model [54, 124] and has also been identified in other lines of APP transgenic mice, including APP/PS1 and TgCRND8 mice [13, 152], as well as in humans with AD [54]. Because genetic ablation of BACE1 also causes Na_V_1.1 depletion [153], one might speculate that the BACEi treatment simply replaced one cause of Na_V_1.1 depletion with another. However, we consider this explanation unlikely because the BACEi did not alter Na_V_1.1 levels in WT controls. Resolving why inhibition of BACE1 failed to block hAPP/Aβ-dependent Na_V_1.1 depletion will likely require a better understanding of the precise mechanisms that result in this abnormality in the context of AD, which are unknown. In addition, other molecular pathways may also be affected by alterations in APP metabolism or protein-protein interactions caused by autosomal dominant AD mutations or increased expression of APP.

## Conclusions

Although FAD mutations, and AD in general, are widely thought to erode brain functions by promoting the cerebral accumulation of Aβ [8, 151, 154], our results highlight the complexity of APP and its potential roles in the pathogenesis of AD, as well as the challenges involved in trying to ascribe functional abnormalities to specific APP metabolites. They also caution against targeting specific APP metabolites for the treatment of AD before the pathobiology of APP is more completely understood.

## Supplementary information

**Additional File 1: Figure S1.** Comparison of APP levels in I5, J20 and KI lines and of Aβ levels in J20 and *App*^NL-F^ lines. (**a**) Cortical and hippocampal levels of APP were determined in 5–6-month-old genetically modified mice from the indicated lines as described in Fig. 1, except that samples from different brain regions were compared on the same western blot. *n* = 3 mice per group. (**b**) Aβ levels in J20 mice and *App*^NL-F^ knock-in mice. Overall levels of Aβ_1-x_ and Aβ_1–42_ in the hippocampus and cortex were determined by ELISA in mice of the indicated genotypes at 2 months of age. *n* = 7–8 mice per group. *P < 0.05, **P < 0.01, ****P < 0.0001 based on repeated measures two-way ANOVA with Holm-Sidak correction (**a**) or multiple Welch *t-*tests with Holm-Sidak correction (**b**). Values are means ± SEM. **Figure S2.** Specificity of the Aβ oligomer assay. (**a**) hAPP and its metabolites, including Aβ and C-terminal fragments (CTFs), are shown together with epitopes recognized by antibodies used in this study. The Aβ_1–42_ sequence within hAPP is shown in blue. 3D6 and 82E1 are specific for the free N-terminus of Aβ, whereas 26D6 recognizes both Aβ and hAPP. 8E5 is an anti-hAPP antibody that binds to the N-terminal region of the protein. CT15 and 85,461 recognize CTFs, including β-CTF (also known as C99), which comprises Aβ and the C-terminus of APP. Elements are not drawn to scale. (**b**) In the Aβ oligomers assay, the same antibody is used for antigen capture and detection. An Aβ dimer (left) and higher-order Aβ oligomers (right) generate an electrochemiluminescence (ECL) signal for detection. Amyloid fibrils are removed by ultracentrifugation prior to oligomer detection. Elements are not drawn to scale. (**c**) ECL signal generation in the Aβ oligomers assay requires the presence of assemblies that contain at least two Aβ molecules displaying the same epitope. Peptides used to test the specificity of this assay were: Aβ_1–6_ monomer; Aβ_1–10_ monomer; Aβ_1–17_ monomer; Aβ_1–17_ monomer containing a methionine residue N-terminal to the free aspartate in Aβ as well as an arginine to glycine substitution at residue 5, which is found in the mouse APP sequence; Aβ1–18 covalent dimer; Aβ_1–29_ covalent dimer with N-methyl modifications to increase solubility and tryptophan addition at the C-terminus to facilitate concentration determination; Aβ_1–40_ di-tyrosine crosslinked dimer; and Aβ_1–42_ oligomer preparations. Each peptide preparation was spiked into bovine serum albumin (BSA) solution, followed by serial dilution, detection using 3D6-3D6, 82E1-82E1, or 26D6-26D6 antibody pairs, and signal quantitation by ECL. All three antibody combinations showed specificity for peptides containing at least two similar epitopes, indicating that epitope overlap, or the binding of two antibodies to the same epitope, does not occur over the concentration range tested here. Aβ_1–42_ oligomer concentrations were based on starting Aβ monomer concentrations. Peptide amino acid sequences are shown in Additional File 1: Figure S3. (**d**) Detection of Aβ preparations spiked into BSA (left) or brain homogenate from wildtype (WT) mice (right). Aβ_1–42_ oligomers prepared as described by Fezoui et al. (1–42 oligomer) [83], Aβ_1–40_ oligomers prepared as described by Lambert et al. (1–40 ADDL) [82] but without incubation, Aβ_1–40_ monomers isolated by size-exclusion chromatography (1–40 SEC monomer), Aβ_1–18_ covalent dimer (1–18 dimer), and Aβ_1–17_ peptide (Aβ 1–17) preparations were added to and serially diluted in BSA solution or brain homogenate over the indicated concentration range. Aβ_1–42_ and Aβ_1–40_ oligomer concentrations were based on starting Aβ monomer concentration. Note that Aβ assemblies prepared to contain at least two similar epitopes showed a significantly higher signal than the monomeric species. The Aβ_1–18_ covalent dimer showed poor spike-recovery in brain homogenate. (**e**) Phosphate buffered saline (PBS) (left) and phosphate buffered saline with 1% Triton X-100 (PBST) (right) soluble fractions of cortical and hippocampal homogenates from 17 to 18-month-old WT and J20 mice were subjected to immunoprecipitation with antibodies that bind to Aβ (3D6, 82E1), hAPP (8E5, CT15), or CTFs (3D6, 82E1, CT15) and the supernatants analyzed with the 3D6/3D6 MSD ELISA. ECL signal in WT controls represents background, as demonstrated in Additional File 1: Figure S4e. In both fractions, signal was reduced to background by immunoprecipitation with antibodies to Aβ, but not to hAPP or CTFs. Similar findings were obtained using the 26D6/26D6 MSD ELISA (Additional File 1: Figure S5g). **Figure S3.** Aβ peptides used as controls and standards in Aβ oligomer assay. (**a**) Amino acid sequences of synthesized peptides. Cysteine mutations introduced into Aβ for dimer formation are highlighted in red. Red bars indicate covalent bonds. Underlined residues indicate the following changes: [− 1]-17 R5G monomer, human to mouse mutation as a negative control for antibody binding; 1–18 dimer, glycine was placed at the C-terminus to facilitate chemical synthesis; 1–29 dimer, “Nme” indicates N-methyl backbone modification at valine and phenylalanine to increase solubility; tryptophan (W) was introduced to facilitate concentration determination. (**b**) Characterization of purified Aβ peptides by reversed-phase high pressure liquid chromatography (HPLC) and electrospray ionization (ESI) mass spectrometry. Peptides were analyzed by HPLC using a 5–65% gradient of solvent B (CH_3_CN + 0.08% TFA) vs. solvent A (H_2_O + 0.1% TFA) over 30 min on a 2.1 × 50 mm C4 column, except for the Aβ_1–29_ dimer, which was analyzed on a 4.6 × 150 mm C18 column. UV detection was at 214 nm. Masses of each peptide were determined separately by nano-ESI LC-MS on a Thermo Fisher Scientific Orbitrap mass spectrometer. **Figure S4.** Effects of tissue homogenization and fractionation on Aβ oligomer measurements and comparison of negative controls. (**a**) Signals in the 3D6/3D6 MSD ELISA were not significantly altered by addition of 1% Triton X-100 to brain homogenates. Synthetic Aβ1–42 oligomers were spiked into hippocampal (left) or cortical (right) brain homogenates prepared with either PBS or PBS + 1% Triton X-100 (PBST). Aβ oligomers were then serially diluted with the respective brain homogenate, followed by analysis with the 3D6/3D6 MSD ELISA. Concentrations are based on starting concentrations of Aβ monomers. (**b**) Tissue fractionation analysis. Cortex from three WT mice (1–3) was homogenized first in PBS and then in PBST. After homogenization with each buffer, the homogenate was centrifuged and the supernatant removed for western blot analysis of proteins that would be expected to be predominantly membrane-bound, soluble, extracellular (EC), or intracellular (IC). Na_V_, sodium channel; NR1, N-methyl-D-aspartate receptor subunit NR1; EAAT2, excitatory amino acid transporter 2. (**c**) Signals in the 3D6/3D6 MSD ELISA were not significantly affected by the tissue homogenization method. Brain tissues were dissected from both hemibrains of 3–4- and 18–20-month-old J20 mice (*n* = 3 per group) and homogenized in PBS buffer using a dounce (left hippocampus + cortex) or blender (right hippocampus + cortex). After ultracentrifugation, the supernatants were analyzed by 3D6/3D6 MSD ELISA. n.s., not significant by unpaired *t* test with Welch correction. mo, months. (**d**) Correlation of Aβ oligomer levels in left and right hippocampi. Aβ oligomer levels in PBS fractions from the left versus right hippocampus of 3–4-month-old J20 mice (*n* = 10) were measured by 3D6/3D6 MSD ELISA. Pearson correlation was used to assess the relationship between measurements in the left and right hippocampi of the same brains. (**e**) Comparison of negative and positive controls for the 3D6/3D6 MSD ELISA. Negative controls included mice that were WT (*App*^+/+^) or homozygous knockout for the endogenous mouse *App* gene (KO, *App*^−/−^), WT mice (−) from each of the genetically modified lines, and J20 samples measured in the absence (No) of the detection (Det) or capture (Cap) antibody (Ab). Samples from J20 and KI mice served as positive controls. Cortical PBST fractions were obtained at 2–3 (I5, J20, KI) or 8 (KO) months of age and analyzed by 3D6/3D6 MSD ELISA. *n* = 5–6 mice per group. ***P < 0.001, ****P < 0.0001 based on one-way ANOVA with Holm-Sidak correction. Some error bars in (A) are too small to visualize. Values in (A, C, E) are means ± SEM. **Figure S5.** Comparison of positive controls for Aβ oligomer assay and validation with additional capture/detection antibody. (**a**) Size-exclusion chromatography (SEC) of Aβ oligomers and peptide standards. Oligomers of recombinant Aβ1–40 (ADDLs) were prepared in vitro as described [82] and purified from monomers by size-exclusion chromatography. Two distinct peptide peaks were observed: a low and a high apparent molecular weight peak. The size of the two species was estimated by comparison with globular protein and sparsely-branched dextran standards [84–86] (elution volume indicated by arrows). These standards showed different relationships between molecular weight and elution on SEC, most likely because of their different hydrodynamic radii. Using these standards and the peptide standards shown in (B), the low molecular weight peak was identified as Aβ_1–40_ monomers. mAU, milli-absorbance units. (**b**) SEC of peptide standards: a peptide consisting of the first 17 amino acids of Aβ (top), a synthetic dimer prepared from the same amino acids except for the two C-terminal residues (middle), and an Aβ_1–40_ monomer (bottom, middle peak). The peaks present at the end of the chromatogram for the 1–18 dimer are detector artifacts and occur after the DMSO peak observed in the chromatogram for the Aβ_1–40_ monomer. A very similar pattern to Aβ_1–40_ was observed for Aβ_1–42_ (data not shown). (**c**) Aβ preparations that were (SEC) or were not purified by SEC were spiked and diluted in brain homogenate, and the oligomer signal was measured with the 3D6/3D6 MSD ELISA. At higher concentrations, Aβ_1–42_ monomer signals were larger than Aβ_1–40_ monomer signals, most likely because of the greater propensity of Aβ_1–42_ monomers to aggregate under such conditions. The SEC-purified oligomers showed higher signals than the unpurified oligomers likely due to the absence of monomeric peptide, which might occupy capture antibody binding sites and lead to reduced signals. (**d**) Brain homogenate from J20 mice was serially diluted with WT brain homogenate, and the oligomer signal measured with the 3D6/3D6 MSD ELISA. ND, no dilution. (**e**) hAPP binding activity of antibodies tested in the Aβ oligomer assay. Cortex plus hippocampus from young (3 months) and old (20 months) WT (−) and J20 (+) mice were homogenized by dounce (PBS fraction) or blender (PBST fraction). The supernatants were analyzed by western blotting using the antibody indicated on the right as the primary antibody. Exposures were similar across blots. Both 6E10 and 26D6 showed strong binding to hAPP (~ 100 kD), whereas 82E1 showed very weak binding to hAPP and 3D6 did not bind to hAPP at all. mo, months. (**f**) Western blot analysis of immunoprecipitated species. Antigens immunoprecipitated from brain homogenates with the antibodies listed on the right were analyzed by western blotting for hAPP and C-terminal fragments using a combination of 6E10 and 82E1 antibodies for detection of hAPP, and CT15 for detection of C-terminal fragments. Aβ is not shown because it was not detected in this experiment. Control conditions lacked the precipitating antibody. Input, brain homogenate; sham, brain homogenate with beads; elut, proteins eluted from sham (Control, left) or from actual immunoprecipitates (Antibody, right); sup, brain homogenate supernatant after immunoprecipitation. (**g**) PBS (left) and PBST (right) soluble fractions of hippocampal plus cortical homogenates from a 17–18-month-old J20 mouse and and an age-matched WT control were subjected to immunoprecipitation with antibodies that bind to Aβ (82E1, 26D6), hAPP (26D6, CT15, 85,461), or C-terminal APP fragments (CTFs) (82E1, 26D6, CT15, 85,461). The supernatants were analyzed with the 26D6/26D6 MSD ELISA. ECL signal in the WT controls represents background. ECL signal in WT controls represents background, as demonstrated in Additional File 1: Figure S4e. In samples from the hAPP mouse, signals were reduced to background by immunoprecipitation with antibodies to Aβ, but not to hAPP or CTFs. Values in (D) are means ± SEM. **Figure S6.** Comparable swim speeds in J20 mice and WT controls treated with BACEi or placebo. J20 mice and WT controls were treated with a BACEi (NB-360) or placebo starting at 1 month of age and continuing throughout behavioral testing at 4–6 months of age. Shown here are swim speeds measured during the cued component of the Morris water maze test. *n* = 12–17 male mice per group. Two-way ANOVA revealed no significant differences among groups. Values are means ± SEM. **Figure S7.** BACEi treatment prevented plaque formation and plaque-associated microgliosis in behaviorally tested J20 mice. J20 mice were treated with the BACEi NB-360 (+) or placebo (−) from 1 month of age until they were sacrificed at 7–8 months of age. All mice underwent behavioral testing as described in Fig. 8. (**a**) Representative coronal sections of the hippocampus that were stained with Thioflavin S (green), labeled with an antibody to the microglial marker Iba1 (magenta), and imaged by confocal microscopy. The higher-magnification insets on the right show multiple activated microglial cells surrounding a Thioflavin S-positive amyloid plaque (top) and a microglial cell in a section lacking such plaques (bottom). The brightness of the Thioflavin S signal in the top inset was reduced to allow for better visualization of the microglial processes. Space bars: 200 μm, 10 μm (Inset). (**b**–**e**) Quantitative assessment of microglia in plaque-free areas of the CA1 region. (**b**) Size of microglia expressed as area occupied by individual Iba1-positive cells. (**c**) Number of Iba1-positive cells per optical field (~100 mm^2^). (**d**) Number of processes (end points) per Iba1-positive cell. (**e**) Length of processes of Iba1-positive cells. Data in (**b**, **d**, **e**) are based on measurements made in ~120 cells from 3 sections per mouse. Data in (**c**) are based on measurements made in 1 field per section and 3 sections per mouse. *n* = 5–11 male mice per group. Two-way ANOVA did not reveal significant differences among groups. Values are means ± SEM. **Figure S8.** Comparison of Zbtb20 and Na_V_1.1 levels in the parietal cortex of J20 and *Zbtb20*^+/−^ mice. Zbtb20 and Na_V_1.1 levels in the parietal cortex were determined by western blot analysis in hemizygous transgenic mice from line J20, *Zbtb20*^+/−^ mice and WT controls from each line at 2 months of age. (**a**) The Zbtb20 antibody (Table S1) was validated by western blot analysis of cortical tissues from 3-week-old *Zbtb20*^+/+^, *Zbtb20*^+/−^ and *Zbtb20*^−/−^ mice. The antibody detects multiple bands in WT controls that are absent in homozygous knockout mice and is sensitive enough to detect partial reductions of Zbtb20 in hemizygous knockout mice. βIII-tubulin was used as a loading control. (**b**) Western blots depicting Zbtb20 and tubulin signals in J20 (top) and *Zbtb20*^+/−^ (bottom) mice and in WT controls from each of these lines. (**c**) Quantitations of relative Zbtb20 levels. Levels in genetically modified mice were expressed relative to the mean Zbtb20/tubulin ratio in WT controls from the respective line, which were arbitrarily defined as 1.0. (**d**) Western blots depicting Na_V_1.1 and tubulin signals in J20 (top) and *Zbtb20*^+/−^ (bottom) mice and in WT controls from each of these lines. (**e**) Quantitations of relative Na_V_1.1 levels. Levels in genetically modified mice were expressed relative to the mean Na_V_1.1/tubulin ratio in WT controls from the respective line, which was arbitrarily defined as 1.0. *n* = 12–16 mice per group. ***P < 0.001, ****P < 0.0001 vs. WT, based on unpaired two-tailed Student t-test. Non-significant (ns) *P* values were 0.0705 (**c**) and 0.8256 (**e**), respectively. Values are means ± SEM.

## Data Availability

All data supporting this study are available from the corresponding author upon reasonable request.

## Abbreviations

Aβ: Amyloid-β
AD: Alzheimer’s disease
APA: Active place avoidance
APP: Amyloid precursor protein
BSA: Bovine serum albumin
CTF: C-Terminal fragments
DAB: Diaminobenzidine
EC: Extracellular
ECL: Electrochemiluminescence
EEG: Electroencephalographic
EPM: Elevated plus maze
ESI: Electrospray ionization
FAD: Familial Alzheimer’s disease
FFT: Fast Fourier transform
hAPP: Human amyloid precursor protein
HBTU: Hexafluorophosphate
HPLC: High pressure liquid chromatography
IC: Intracellular
MSD: Meso Scale Discovery
MWM: Morris water maze
NPY: Neuropeptide Y
OF: Open field
PBS: Phosphate-buffered saline
PBST: Phosphate buffered saline with Triton X-100
SEC: Size-exclusion chromatography
SWDs: Spike-and-wave discharges
WT: Wildtype
