# Supplementary material for: Behavioral and neural network abnormalities in human APP transgenic mice resemble those of App knock-in mice and are modulated by familial Alzheimer’s disease mutations but not by inhibition of BACE1
Source: Mol Neurodegener. 2020 Sep 14;15:53. doi: 10.1186/s13024-020-00393-5 (PMC7489007; doi:10.1186/s13024-020-00393-5)
Supplement: Supplementary file 1 — Additional File 1: Figure S1. Comparison of APP levels in I5, J20 and KI lines and of Aβ levels in J20 and AppNL-F lines. (a) Cortical and hippocampal levels of APP were determined in 5–6-month-old genetically modified mice from the indicated lines as described in Fig. 1, except that samples from different brain regions were compared on the same western blot. n = 3 mice per group. (b) Aβ levels in J20 mice and AppNL-F knock-in mice. Overall levels of Aβ1-x and Aβ1–42 in the hippocampus and cortex were determined by ELISA in mice of the indicated genotypes at 2 months of age. n = 7–8 mice per group. *P < 0.05, **P < 0.01, ****P < 0.0001 based on repeated measures two-way ANOVA with Holm-Sidak correction (a) or multiple Welch t-tests with Holm-Sidak correction (b). Values are means ± SEM. Figure S2. Specificity of the Aβ oligomer assay. (a) hAPP and its metabolites, including Aβ and C-terminal fragments (CTFs), are shown together with epitopes recognized by antibodies used in this study. The Aβ1–42 sequence within hAPP is shown in blue. 3D6 and 82E1 are specific for the free N-terminus of Aβ, whereas 26D6 recognizes both Aβ and hAPP. 8E5 is an anti-hAPP antibody that binds to the N-terminal region of the protein. CT15 and 85,461 recognize CTFs, including β-CTF (also known as C99), which comprises Aβ and the C-terminus of APP. Elements are not drawn to scale. (b) In the Aβ oligomers assay, the same antibody is used for antigen capture and detection. An Aβ dimer (left) and higher-order Aβ oligomers (right) generate an electrochemiluminescence (ECL) signal for detection. Amyloid fibrils are removed by ultracentrifugation prior to oligomer detection. Elements are not drawn to scale. (c) ECL signal generation in the Aβ oligomers assay requires the presence of assemblies that contain at least two Aβ molecules displaying the same epitope. Peptides used to test the specificity of this assay were: Aβ1–6 monomer; Aβ1–10 monomer; Aβ1–17 monomer; Aβ1–17 monomer containing a methi [file 13024_2020_393_MOESM1_ESM.pdf]

Table S1 Antibodies Used

PRIMARY						
**Antigen**	**Antibody Name***	**Host Species**	**Source**	**Catalog #**	**Assay**	**Dilution**
$A\beta_{1–5}$	3D6	Mouse	Janssen or from ATCC hybridoma cell line	PTA-5130	WB<br>IHC<br>ELISA	1:1,000<br>1:3,000<br>2 µg/ml
$A\beta_{1–12}$	26D6	Mouse	Bristol-Myers Squibb		WB<br>ELISA	1:1,000<br>2 µg/ml
$A\beta_{1–16}$	82E1	Mouse	IBL	10326	WB	1:1,000
$A\beta_{3–8}$	6E10	Mouse	BioLegend	803001	WB	1:1,000
$A\beta_{13–28}$	266	Mouse	Janssen		ELISA	10 µg/ml
$A\beta_{33–42}$	21F12	Mouse	Janssen		ELISA	5 µg/ml
Actin α1	Actin	Rabbit	Sigma-Aldrich	A2066	WB	1:3,000
APP A4 (N-Term)	22C11	Mouse	EMD Millipore	MAB348	WB	1:1,000
Human APP	8E5	Mouse	Janssen		WB	1:10,000
APP (CTFs)	CT15<br><br>85461	Rabbit<br><br>Mouse	Edward Koo, UCSD<br><br>Bristol-Myers Squibb		WB<br><br>WB	1:2,000<br><br>1:1,000
Calbindin		Rabbit	Swant	CB38	IHC	1:30,000
c-Fos [2H2]		Mouse IgG1	Abcam	ab208942	IHC	1:2,000
EAAT2		Rabbit	Abcam	ab41621	WB	1:3,000
GAPDH		Mouse	Millipore	MAB374	WB	1:10,000
Iba1		Rabbit	Wako	019-19741	IHC	1:1000
$Na_V1.1$		Rabbit	Alomone Labs	ASC-001	WB	1:1,000
$Na_V1.2$		Rabbit	Alomone Labs	ASC-002	WB	1:1,000
$Na_V1.6$		Rabbit	Alomone Labs	ASC-009	WB	1:1,000
Neuropeptide Y		Rabbit	ImmunoStar	22940	IHC	1:2,000
NR1		Mouse	EMD Millipore	05-432	WB	1:1,000
Pan Voltage-gated Sodium Channel, α subunit	$PanNa_v$	Rabbit	Sigma-Aldrich	S6936	WB	1:1,000
Parvalbumin		Rabbit	Swant	PV27	WB	1:5,000

Spondin-1	SPON1	Rabbit	Abcam	ab40797	WB	1:1,000
βIII-Tubulin		Rabbit	Abcam	ab18207	WB	1:3,000
Zbtb20		Rat	BD Biosciences	565453	WB	1:1,000
**SECONDARY**						
**Antibody Name**	**Conjugated Tag**	**Host Species**	**Source**	**Catalog #**	**Assay**	**Dilution**
Anti-Mouse	Alexa Fluor 546	Donkey	Life Technologies	A10036	IHC	1:500
	Biotin-SP	Goat	Jackson ImmunoResearch	115-067-003	IHC	1:500
	IRDye 680LT	Goat	LI-COR	925-68020	WB	1:10,000
	IRDye 800CW	Donkey	LI-COR	925-32212	WB	1:10,000
Anti-Rabbit	Biotin-SP	Donkey	Jackson ImmunoResearch	711-065-152	IHC	1:1,000
	HRP	Goat	Calbiochem	401515	WB	1:5,000
	IRDye 680LT	Goat	LI-COR	925-68021	WB	1:10,000
	IRDye 800CW	Donkey	LI-COR	926-32213	WB	1:10,000
Anti-Rat	HRP	Goat	Millipore	AP136P	WB	1:1,000

*left blank if synonymous with the antigen that the antibody recognizes

Figure S1

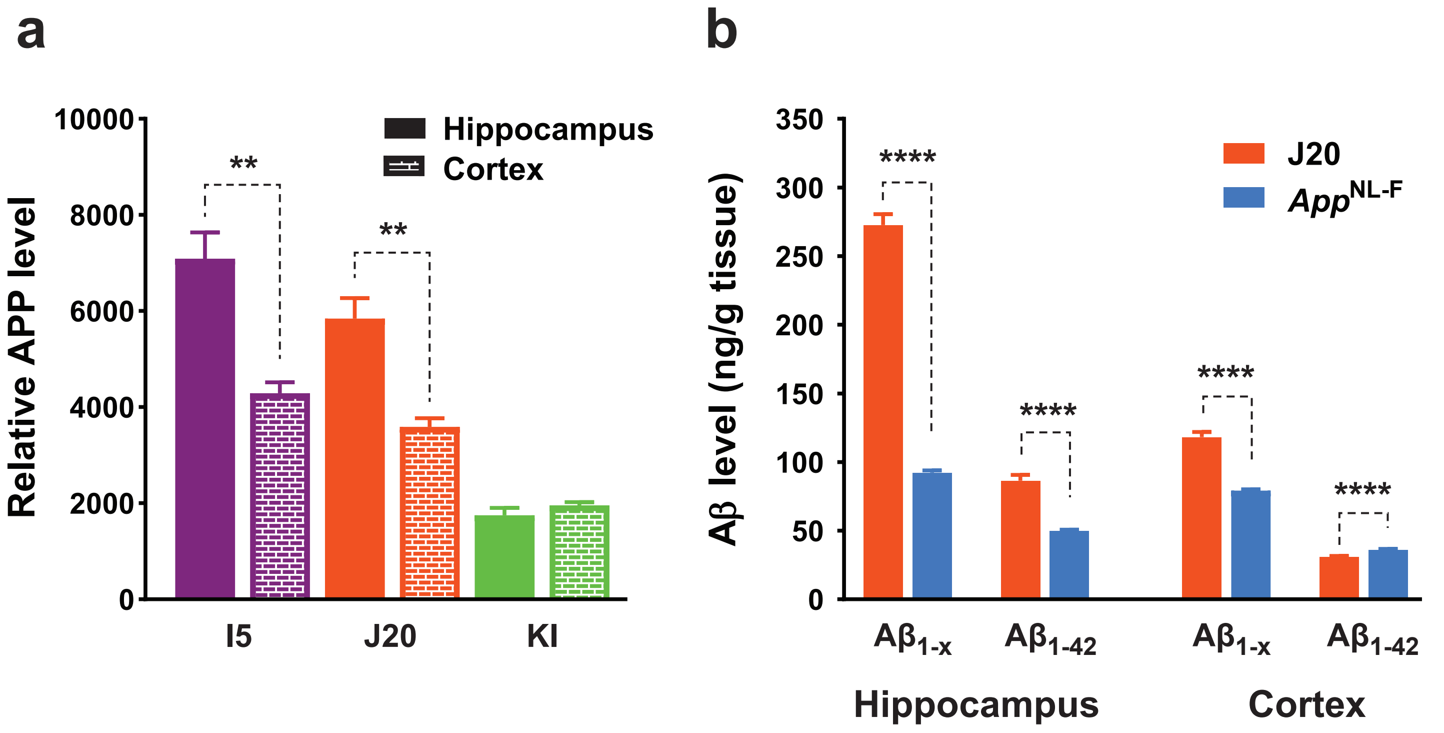

a
10000
8000
6000
4000
2000
0
Relative APP level
Hippocampus
Cortex
**
**
I5
J20
KI
b
350
300
250
200
150
100
50
0
Aβ level (ng/g tissue)
J20
App^NL-F
****
****
****
****
Aβ1-x
Aβ1-42
Aβ1-x
Aβ1-42
Hippocampus
Cortex

Figure S2

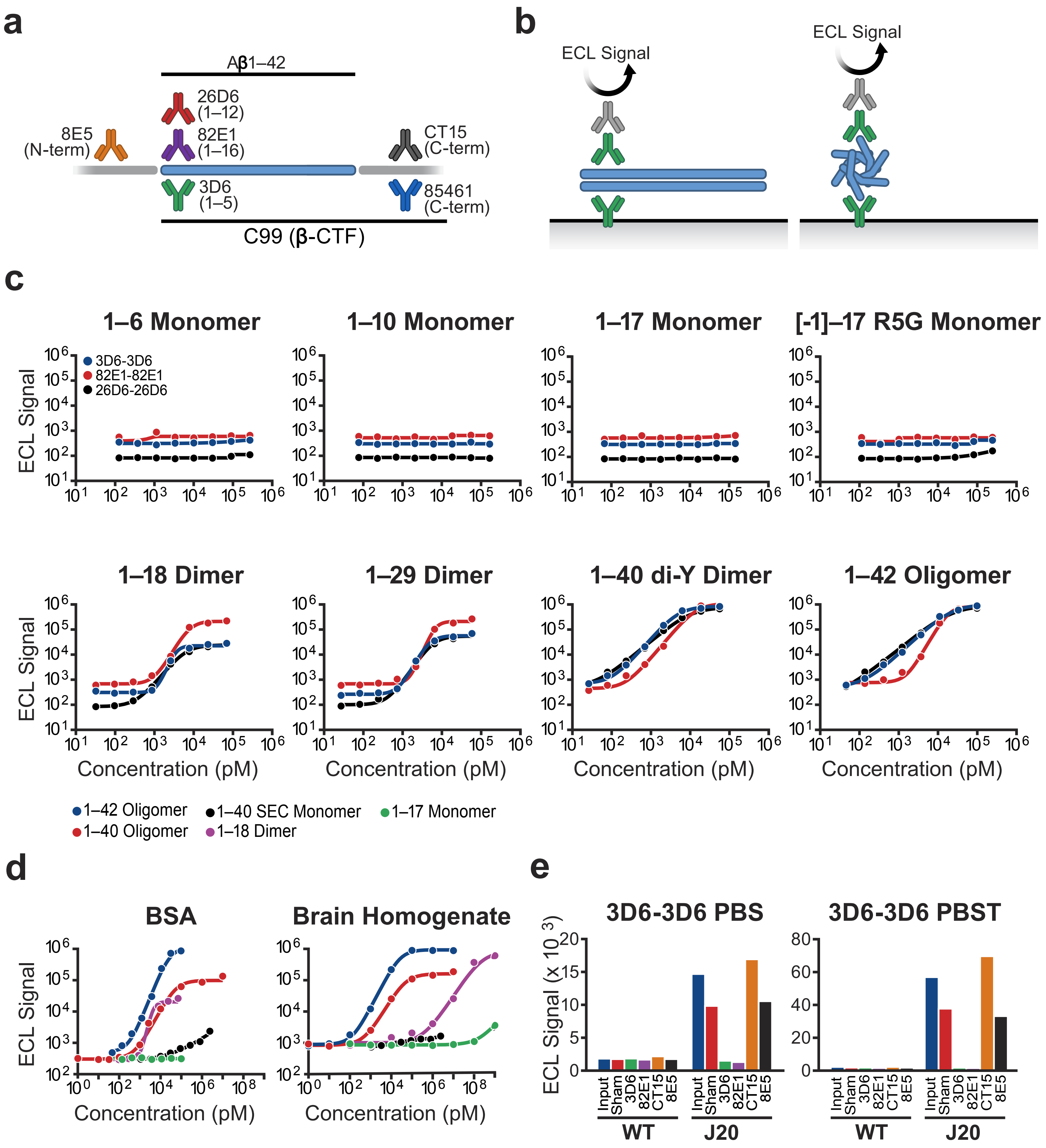
a
Aβ1–42
26D6
(1–12)
82E1
(1–16)
8E5
(N-term)
CT15
(C-term)
3D6
(1–5)
85461
(C-term)
C99 (β-CTF)
b
ECL Signal
ECL Signal
c
1–6 Monomer
1–10 Monomer
1–17 Monomer
[-1]–17 R5G Monomer
3D6-3D6
82E1-82E1
26D6-26D6
ECL Signal
1–18 Dimer
1–29 Dimer
1–40 di-Y Dimer
1–42 Oligomer
ECL Signal
Concentration (pM)
Concentration (pM)
Concentration (pM)
Concentration (pM)
1–42 Oligomer
1–40 SEC Monomer
1–17 Monomer
1–40 Oligomer
1–18 Dimer
d
BSA
Brain Homogenate
ECL Signal
Concentration (pM)
Concentration (pM)
e
3D6-3D6 PBS
3D6-3D6 PBST
ECL Signal (x 10 3)
Input
Sham
3D6
82E1
CT15
8E5
WT
J20

Figure S3

a

DAEFRH	**1–6 monomer**
DAEFRHDSGY	**1–10 monomer**
DAEFRHDSGYEVHHQKL	**1–17 monomer**
MDAEF GHDSGYEVHHQKL	**[−1]–17 R5G monomer**
DAEFRHDSGYEVHHQKCG<br>DAEFRHDSGYEVHHQKCG	**1–18 dimer**
DAEFRHDSGYEVHHQKLV(Nme) FF(Nme) AEDVGCNWG<br>DAEFRHDSGYEVHHQKLV(Nme) FF(Nme) AEDVGCNWG	**1–29 dimer (chemically-modified)**
DAEFRHDSGYEVHHQKLVFFAEDV GSNKGAIIGLMVGGVV	**1–40**
DAEFRHDSGYEVHHQKLVFFAEDVGSNKGAIIGLMVGGVV<br>DAEFRHDSGYEVHHQKLVFFAEDVGSNKGAIIGLMVGGVV	**1–40 di-Y dimer**
DAEFRHDSGYEVHHQKLVFFAEDV GSNKGAIIGLMVGGVVIA	**1–42**

b

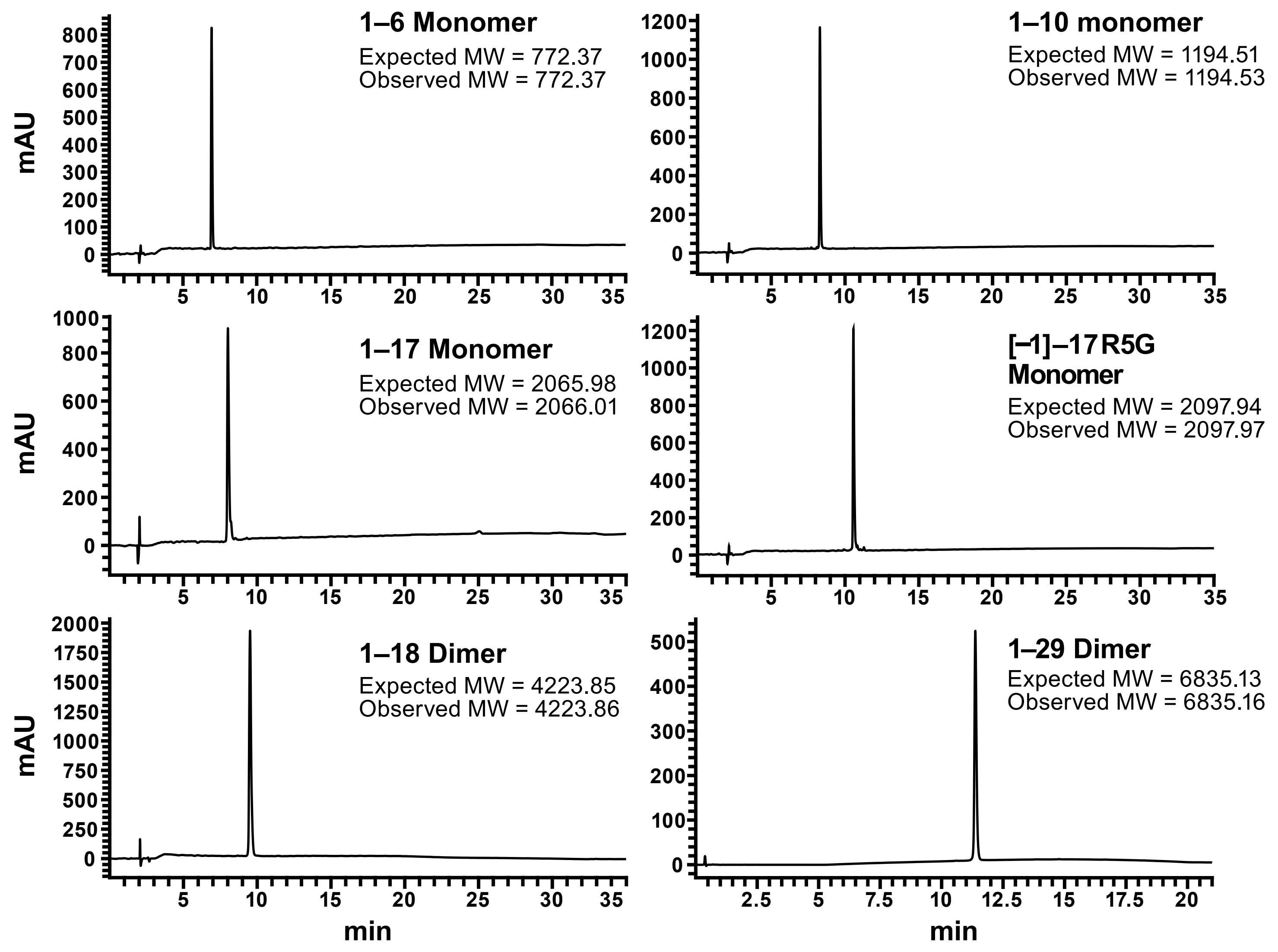

Figure S4

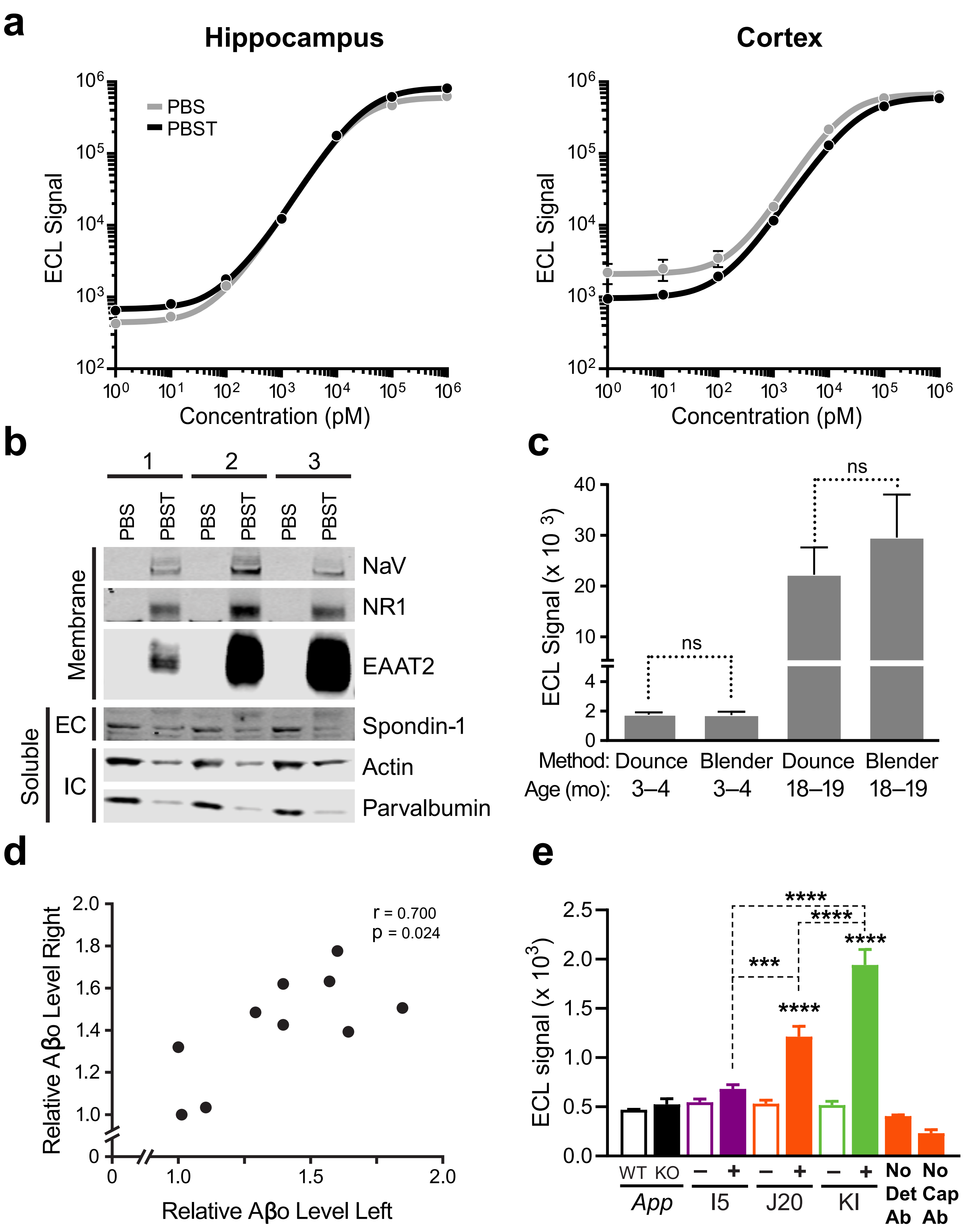
a
Hippocampus
Cortex
PBS
PBST
ECL Signal
Concentration (pM)
b
1
2
3
PBS
PBST
Membrane
Soluble
EC
IC
NaV
NR1
EAAT2
Spondin-1
Actin
Parvalbumin
c
ECL Signal (x 10 3)
ns
Method: Dounce Blender Dounce Blender
Age (mo): 3–4 3–4 18–19 18–19
d
r = 0.700
p = 0.024
Relative Aβo Level Right
Relative Aβo Level Left
e
ECL signal (x 10^3)
WT KO
App
I5
J20
KI
No Det Ab
No Cap Ab

Figure S5

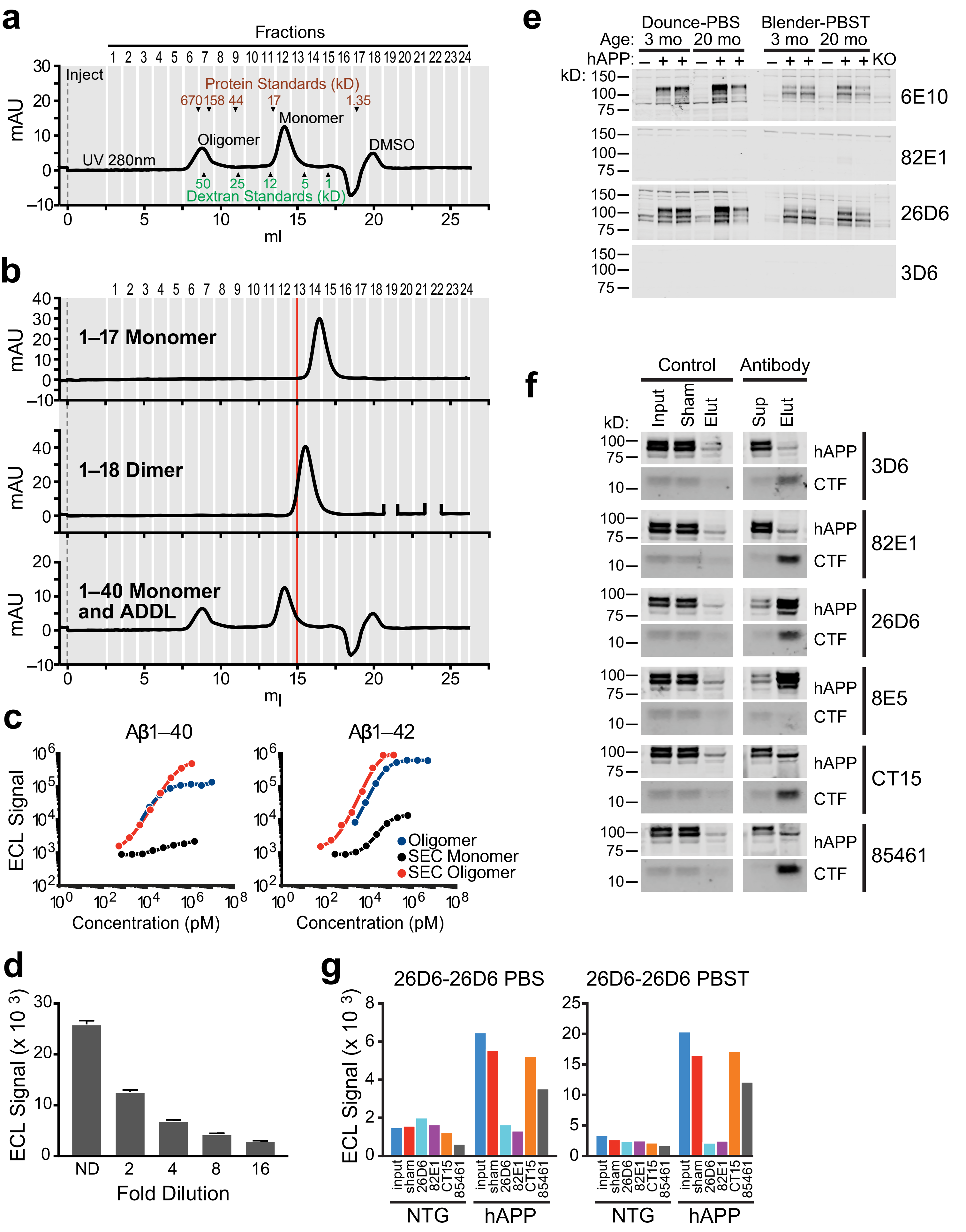

a
Fractions
Inject
Protein Standards (kD)
670 158 44 17 1.35
Monomer
Oligomer
DMSO
UV 280nm
50 25 12 5 1
Dextran Standards (kD)
mAU
ml
b
1–17 Monomer
1–18 Dimer
1–40 Monomer and ADDL
mAU
ml
c
Aβ1–40
Aβ1–42
ECL Signal
Concentration (pM)
Oligomer
SEC Monomer
SEC Oligomer
d
ECL Signal (x 10 3)
ND 2 4 8 16
Fold Dilution
e
Dounce-PBS
Blender-PBST
Age: 3 mo 20 mo 3 mo 20 mo
hAPP: – + + – + + – + + – + + KO
kD:
6E10
82E1
26D6
3D6
f
Control
Antibody
Input Sham Elut Sup Elut
kD:
hAPP
CTF
3D6
82E1
26D6
8E5
CT15
85461
g
26D6-26D6 PBS
26D6-26D6 PBST
ECL Signal (x 10 3)
input sham 26D6 82E1 CT15 85461
NTG
hAPP

Figure S6

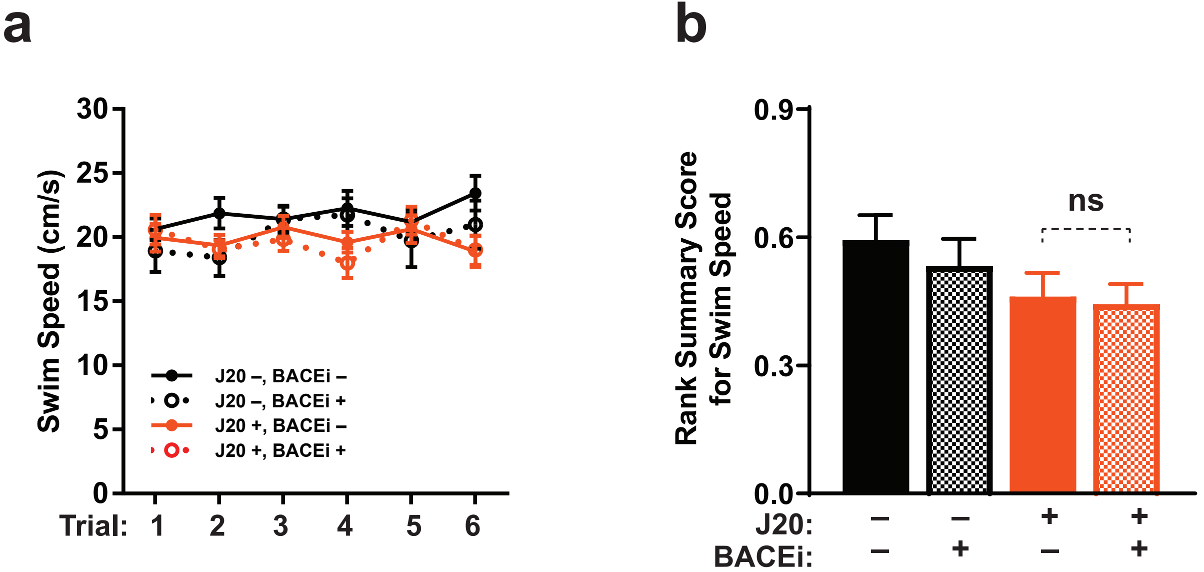
a
30
25
20
15
10
5
0
Swim Speed (cm/s)
Trial:
1
2
3
4
5
6
J20 –, BACEi –
J20 –, BACEi +
J20 +, BACEi –
J20 +, BACEi +
b
0.9
0.6
0.3
0.0
Rank Summary Score for Swim Speed
ns
J20: – – + +
BACEi: – + – +

Figure S7

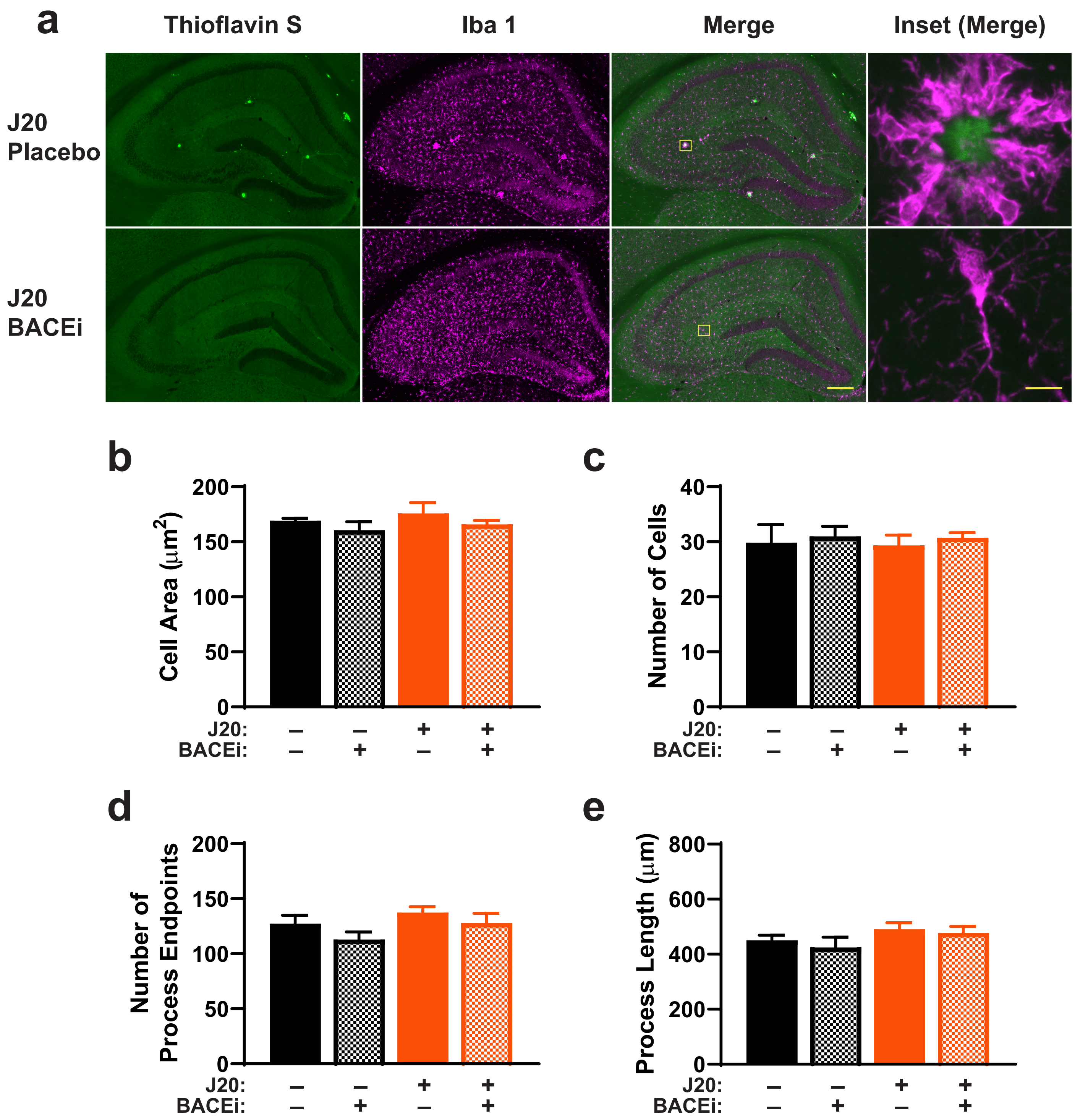
a
Thioflavin S
Iba 1
Merge
Inset (Merge)
J20 Placebo
J20 BACEi
b
Cell Area (μm2)
200
150
100
50
0
J20: – – + +
BACEi: – + – +
c
Number of Cells
40
30
20
10
0
J20: – – + +
BACEi: – + – +
d
Number of Process Endpoints
200
150
100
50
0
J20: – – + +
BACEi: – + – +
e
Process Length (μm)
800
600
400
200
0
J20: – – + +
BACEi: – + – +

Figure S8

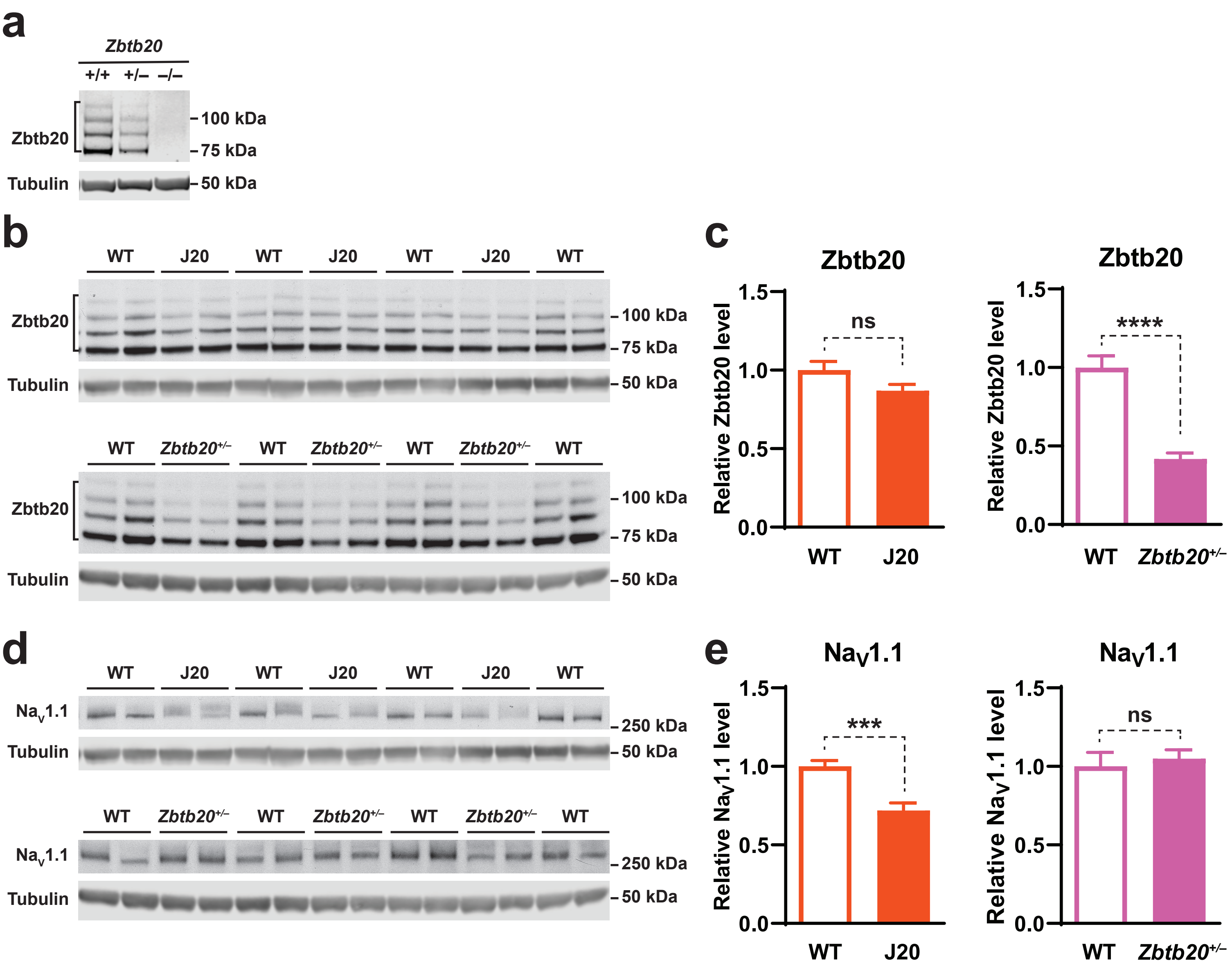
a
Zbtb20
+/+ +/– –/–
Zbtb20
100 kDa
75 kDa
Tubulin
50 kDa
b
WT J20 WT J20 WT J20 WT
Zbtb20
100 kDa
75 kDa
Tubulin
50 kDa
WT Zbtb20+/– WT Zbtb20+/– WT Zbtb20+/– WT
Zbtb20
100 kDa
75 kDa
Tubulin
50 kDa
c
Zbtb20
Relative Zbtb20 level
1.5
1.0
0.5
0.0
ns
WT
J20
Zbtb20
Relative Zbtb20 level
****
WT
Zbtb20+/–
d
WT J20 WT J20 WT J20 WT
Nav1.1
250 kDa
Tubulin
50 kDa
WT Zbtb20+/– WT Zbtb20+/– WT Zbtb20+/– WT
Nav1.1
250 kDa
Tubulin
50 kDa
e
Nav1.1
Relative Nav1.1 level
1.5
1.0
0.5
0.0
***
WT
J20
Nav1.1
Relative Nav1.1 level
ns
WT
Zbtb20+/–
